# Individual child factors affecting the diagnosis of attention deficit hyperactivity disorder (ADHD) in children and adolescents: a systematic review

**DOI:** 10.1007/s00787-024-02590-9

**Published:** 2024-10-07

**Authors:** Lok Yee Chloe Tam, Yanisa Taechameekietichai, Jennifer L. Allen

**Affiliations:** 1 Department of Psychology, University of Bath, 10 West, Claverton Down, Bath, BA2 7AY UK; 2 University Hospitals Sussex NHS Foundation Trust, Worthing, UK

**Keywords:** ADHD, Diagnosis, Clinical Factors, Socio-demographic factors, Child

## Abstract

Attention deficit hyperactivity disorder (ADHD) is a prevalent condition characterised by patterns of inattention, hyperactivity, and impulsivity. This systematic literature review aims to identify the child clinical and sociodemographic factors related to the detection and diagnosis of ADHD in children and adolescents, given concerns about delays or failures in identifying ADHD as well as over-diagnosis, hindering appropriate and timely support for children and families. Through electronic and manual searches of peer-reviewed and grey literature, 5852 articles were identified and subjected to two stages of screening by independent reviewers. Due to the heterogeneity in study methods, a narrative approach was used to summarise study findings. Forty-one studies meeting eligibility criteria revealed sixteen child-related factors influencing the ADHD diagnostic process. These factors include ADHD subtype, ADHD symptom severity, comorbid mental disorders, behavior problems, internalizing symptoms, functional impairment, social and cognitive functioning, physical health, gender, age, relative age, race/ethnicity, socio-economic status, insurance coverage, residence urbanicity, and family structure. While the impact of many of these factors on ADHD diagnosis was inconsistent due to the substantial diversity in study designs and context, certain patterns emerged. Meeting the criteria for the inattentive subtype, lower functional impairment, female gender, Black or Latinx ethnicity, and being relatively young for their grade were consistently found to be associated with a delayed or absent ADHD diagnosis. Further research is needed to explore the intricate relationships between these child-level variables and their implications for ADHD recognition, referral, and evaluation, especially outside the USA.

**Supplementary Information:**

The online version contains supplementary material available at 10.1007/s00787-024-02590-9.

## Introduction

Attention deficit hyperactivity disorder (ADHD) is a neurodevelopmental condition characterised by three core symptoms: persistent and excessive levels of inattention, hyperactivity and impulsivity [1, 2]. The Diagnostic and Statistical Manual of Mental Disorders (DSM-V) [1, 2] currently classifies ADHD into three primary subtypes: predominantly inattentive (ADHD-I), predominantly hyperactive-impulsive (ADHD-HI), and a combined subtype (ADHD-C). Although ADHD is associated with a wide range of psychosocial difficulties and impairment [3], it is increasingly recognized that children with ADHD are neurodivergent, with ADHD-related traits existing on a spectrum. Aspects of ADHD can be adaptive rather than impairing, with positive traits associated with ADHD including cognitive dynamism, non-conformism, creativity, and flexibility [4, 5]. Nonetheless, ADHD is often comorbid with other mental disorders such as oppositional defiant disorder (ODD), conduct disorder (CD), anxiety disorders, autism spectrum disorder (ASD), mood disorders, and learning and intellectual difficulties [6, 7]. While acknowledging that there are strengths associated with ADHD, it also predicts a wide range of adverse outcomes, including academic and vocational under-achievement [8], social difficulties [9], proneness to accidents and injuries [10], reduced quality of life [11], increased criminality [12], and increased risk for premature mortality [13]. Therefore, the early and accurate detection of ADHD in children is crucial to mitigate potential negative outcomes.

ADHD is relatively common, estimated to affect 5–8% of school-age children, depending on study sampling methods, location, and the diagnostic instruments used [14–16]. Ideally, all children with ADHD would be identified early on and receive an appropriate diagnosis. However, there are wide variations in ADHD diagnosis rates that cannot be explained by unequal healthcare access or differences in ADHD symptom severity [17], raising concerns about the potential over- or under-diagnosis of ADHD. The process of obtaining an ADHD diagnosis typically involves multiple steps [18]. In the current review, the receipt and timing of the diagnosis of ADHD are understood as encompassing the entire diagnostic process. While the specifics of obtaining an ADHD diagnosis can vary across countries and settings, the process generally follows these steps: the detection of ADHD symptoms, referral for specialist assessment, comprehensive evaluation, and ultimately a formal diagnosis by a clinician. Importantly, diagnosing ADHD involves more than just identifying symptoms; it typically begins with parents or teachers recognising potential problems. Parents may seek initial consultation with a GP, who may then refer the child to a specialist. Similarly, teachers who observe ADHD symptoms can initiate a referral. Specialists then collaborate with parents, educators and the child to conduct a thorough evaluation before making a formal diagnosis, and providing appropriate support if needed. While this review will be confined to child-level factors, it is recognised that these factors both shape and are shaped by their socio-cultural environment. Receipt of an ADHD diagnosis may be the result of a complex interplay between interconnected factors operating across multiple levels, thus findings will be interpreted in relation to the family, school, health and broader systems in which individual child-level factors are embedded [19]. Consistent with the ‘goodness of fit’ model [20], whether or not ADHD traits are adaptive or maladaptive depends on the environmental context. Supportive home, school and work environments can enable children and adolescents with ADHD to express related strengths, mediating and/or compensating for ADHD symptoms that might otherwise cause distress and impairment [5], as such if the threshold for diagnosis is reached, this may not occur until later in life, potentially leading to delayed diagnosis.

Concerns have been expressed regarding the potential delay or failure to identify ADHD in children [e.g., 21–23, as it prevents children and their families from accessing support and achieving their full potential, which may lead to cascading adverse outcomes across domains [3], such as the development of comorbid difficulties such as substance abuse [6]. Dalsgaard et al. [13] reported an elevated premature mortality rate among individuals with a delayed diagnosis of ADHD. The negative impact of ADHD can be diminished by careful monitoring and appropriate interventions, including medication [24], and various non-pharmacological interventions [25, 26]. Receipt of an ADHD diagnosis has also been shown to improve children and families’ understanding of the condition, thus providing them coping and problem-solving skills [27]. With neurodiversity affirming practices and engagement-focused family therapies, an ADHD diagnosis can empower children to strengthen or express aspects of ADHD that are adaptive in some contexts, contributing to the development of positive self-concept [28, 29]. Given the substantial personal, familial, and societal costs of ADHD [30, 31], an increased understanding of the factors influencing the receipt and timing of ADHD diagnosis may help to promote timely and accurate identification, diagnosis and service access.

There is a large gap between the age of onset and age of diagnosis in European children [22], and many children with ADHD remain undiagnosed across many countries [23, 32]. This under-diagnosis appears to be affecting certain groups of children disproportionately, with the child’s socioeconomic status (SES) [33], gender [23, 34], and race/ethnicity [35, 36] affecting the diagnosis of ADHD. A meta-analysis featuring data from 102 studies from all world regions showed that the male-to-female prevalence ratio was higher in clinical (4:1) than population studies (2.4:1) [32]. Girls are estimated to be diagnosed five years later than boys on average [37]. Children from minoritized ethnic and linguistic communities are also less likely to receive a diagnosis than White children, despite having more pronounced symptoms [38, 39].

There is also evidence suggesting that ADHD is over-diagnosed in specific groups of children [38, 40]. Rowland et al. [41] revealed that 10% of elementary students in rural North Carolina have been misdiagnosed with ADHD diagnosis when they did not meet diagnostic criteria. Likewise, within a cohort exhibiting adequate functioning, White children had a greater likelihood of receiving an ADHD diagnosis than children from minoritized ethnic backgrounds [42]. Moreover, children with a more ‘prototypical’ ADHD presentation, such as boys with behavior problems, may be over-diagnosed, even by qualified specialist clinicians [40]. It has been speculated that overdiagnosis could be the result of ADHD-related marketing from pharmaceutical companies [43], the increase in misinformation about ADHD on social media platforms leading to inaccurate self-diagnosis [44], or by the desire for parents, teachers and the medical profession to manage the disruptive behavior of children that fail to live up to social norms, demands and expectations [45].

There is concern over the over-diagnosis of ADHD due to the potential for the unnecessary medicalisation of children [46]. The economic burden of ADHD medication is substantial, and the long-term safety of different medications used to treat ADHD in children is largely unknown [47]. Medications prescribed for ADHD have been linked to weight loss, hepatotoxicity, an increased risk of suicidal ideation [48] and serious cardiovascular events [49]. However, it is important to note that medications are not incentivised for prescribers in most countries outside of the USA, which may contribute to cross-cultural differences in prescription rates. Child-related factors influencing ADHD diagnoses may differ across different settings, countries, and time periods, given different health and social care policies and systems.

Both the underdiagnosis and overdiagnosis of ADHD can present significant challenges. With the heterogenous prevalence rates of ADHD within [e.g., 17, 50 and between countries [51, 52], coupled with high levels of comorbidity with other mental disorders [7], a better understanding of the child sociodemographic and clinical factors contributing to the receipt and timing of ADHD diagnoses is needed. A past review of factors influencing the barriers and facilitators in the pathway towards an ADHD diagnosis [53] was limited to papers published before 2012. There have been advances in the recognition and treatment of ADHD, and with the rise in ADHD prevalence in the past few decades which may be due to increased awareness and detection or overdiagnosis [23, 54], a more updated review is warranted. Kappi and Martel [55] investigated barriers in seeking mental health services for ADHD in children, but focused on factors related to caregiver help-seeking rather than individual child factors that may form a barrier to diagnosis. French et al. [56] reviewed barriers to the recognition of ADHD, but restricted study to factors specific to the primary care setting. Different child characteristics, including age, gender, ethnicity, SES, symptom type and severity, and comorbid diagnoses have been investigated in relation to an ADHD diagnosis, but to our knowledge, no systematic review has been conducted synthesizing these factors. Therefore, the aim of this study is to identify child-level clinical and socio-demographic factors that are related to the identification and diagnosis of ADHD.

### Aim of the current review

The aim of the study is to systematically review evidence for child-related factors associated with an ADHD diagnosis in children and adolescents, encompassing factors that contribute to the delay of or failure to diagnose ADHD, as well as those that facilitate the identification of ADHD. We hypothesized that a wide range of child-level factors, both clinical and socio-demographic variables, would be identified as factors related to the timing and receipt of an ADHD diagnosis. Specifically, we anticipate that male gender, White ethnicity, higher SES, greater symptom severity, impairment, and disruptive behavior will enhance the likelihood of children to receive an ADHD diagnosis or obtain an earlier diagnosis.

## Methods

The systematic review protocol was pre-registered with PROSPERO (registration number: CRD42023392902), and the Preferred Reporting Items for Systematic Reviews and Meta-Analysis (PRISMA) [57] standards were followed at all stages of this review.

### Search methods and strategies

A systematic literature search was developed in consultation with a specialist librarian. The search strategy was extensive to facilitate the identification of all eligible studies. The following electronic databases were searched: PsycINFO (APA PsycNET); MEDLINE (PubMED.gov); Embase (Embase.com); and Educational Resources Information Center (ERIC; EBSCO). To identify unpublished studies, dissertations, pre-prints or grey literature, PsycEXTRA (APA PsycNET) and OpenDissertations (EBSCO) were also searched. The pre-registered protocol was advertised on social media (Twitter, Researchgate) and prominent researchers in the field were contacted to identify any further unpublished studies. A thorough inspection of the reference lists of the included articles was also performed to ensure that all eligible studies were identified.

### Search terms and strategies

A preliminary analysis of the subject headings, as well as the text words in the title and abstract of articles found in the initial search was conducted, and search terms were subsequently adjusted to maximize search sensitivity (see Supplementary Material 1). The search strategy was developed on Embase and subsequently adapted for each database. It involved a combination of identified index terms and free text words in the abstract and/or title and/or subject/keywords/descriptors relating to the following concepts: (1) ADHD; (2) diagnosis/identification; (3) factors/barriers; (4) children/young people. Boolean operators and truncation symbols were used to compile the search strings on each database. An English language filter and publication date limit from 1968 was imposed, as ADHD, or its antecedent conceptualisation, Hyperkinetic Reaction of Childhood, was formally included into the official medical nomenclature in the DSM-II (APA) [58]. The searches also excluded animal studies, target ADHD population aged 18 years or older, as well as publication types that do not include any data. For further details on search methods, see Supplementary Material 1.

### Eligibility criteria

The studies were screened according to the following inclusion criteria: (1) original article, (2) child-level factors, encompassing clinical and socio-demographic factors, that influence the diagnosis of ADHD, where diagnosis is defined as any stage within the diagnostic process of ADHD, (3) included children aged ≤ 18 years with a clinical or research diagnosis of ADHD, and (4) in the English language. We excluded commentaries, opinions, book chapters, conference abstracts, review papers, and case studies from the review (see Supplementary Material 1 for further details of the inclusion and exclusion criteria).

### Data selection

The search was conducted by the first author (LT) in February 2023. All search results were stored in EndNote initially and later imported into Covidence. After duplicates were removed, all identified articles were screened according to the eligibility criteria in two stages, i.e., review based on the titles and abstract, followed by a full-text screen. The first stage was completed by the primary rater (LT), with a random sample of 10% being double-reviewed by a second independent rater (YT). All full-texts were screened independently by both raters. Following each stage, disagreements were resolved through discussion. In the event of uncertainty or when the consensus could not be reached, a third reviewer (JA) was consulted. Cohen’s Kappa (κ) was calculated to assess inter-rater reliability, yielding values of .71 and .76 for the two stages, respectively, indicating substantial agreement between the reviewers.

### Data extraction and quality assessment

A data extraction proforma was designed, piloted and amended by LT, in consultation with JA (see Supplementary Material 1). To reduce the risk of bias, papers were extracted by LT and YT independently, and discrepancies were discussed and resolved afterwards. Data were extracted on the following: (1) Publication details; (2) Country of study; (3) Study aims; (4) Study type; (5) Study design; (6) Participant information; (7) ADHD diagnostic measures; (8) Measures of child-related factors, including the measurement tools, sources and informants, and coding systems; (9) Method of analysis; (10) Summary of relevant key findings, including the child-related factors in question, stages within the diagnostic process affected, the direction of effects and author’s relevant conclusions; and (11) Risk of bias information (strengths and limitations of the study). All relevant findings including main or secondary findings, were extracted and incorporated into the review.

For the quality assessment, the same two independent raters evaluated all the included articles. Considering the anticipated inclusion of diverse study designs, the intended quality assessment tool cited in the study protocol was the Mixed Methods Appraisal Tool (MMAT-2018) [59]. However, none of the eligible studies featured a qualitative or mixed methods design. Therefore, a Risk of Bias in Non-randomized Studies (RoBANS) [60] was used, given its specificity to the evaluation of case–control and cohort studies. The RoBANS was modified to provide a better fit with the included studies (see Supplementary Material 1), e.g., the domain ‘Measurement of exposure’ was changed to ‘Measurement of child-level factors’. The tool evaluates six domains (selection of participants, confounding variables, measurement of child-level factors, blinding of outcome measurements, incomplete outcome data, selective outcome reporting) as ‘high’, ‘low’, or ‘unclear’. It showed fair to moderate inter-rater reliability [60]. The overall study quality were not used as an exclusion criterion to provide a thorough overview of the factors identified in the literature; instead, study quality were referred to when interpreting findings.

### Data synthesis

Given the heterogenous methodological approaches employed in studies retrieved through the literature search, a narrative synthesis was used to analyse the relevant findings. The child-related factors identified were organised based on thematic similarities, then how these factors were related to ADHD diagnosis were summarised descriptively. This strategy enables connections between different study findings, helping to highlight the interplay between various factors and to identify gaps in the literature. For each thematic category, the relationship between the factor and the receipt of diagnosis is addressed first, followed by the timing of the diagnosis. If papers delineated where the factors might influence diagnosis along the diagnostic pathway, this information was presented according to the linear process of the ADHD diagnostic pathway. Details of the methodological approaches and the context of the studies are also reported in instances where discernible patterns have emerged.

## Results

### Study selection and characteristics

**Fig. 1:**
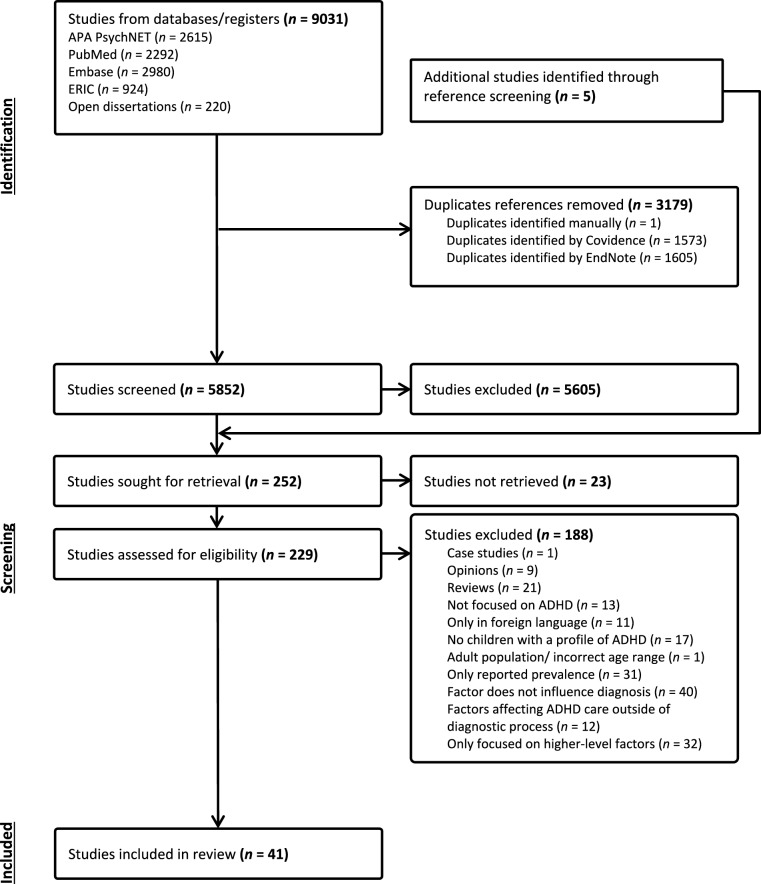
PRISMA flow diagram illustrating the selection process of the systematic review

The study selection process is illustrated in Fig. 1, following PRISMA guidelines [57]. The initial screen yielded 9031 results. After duplicates were removed, 5852 articles were initially screened according to eligibility criteria based on title and abstract, where 5605 records were excluded. Five additional studies were identified through reference screening. As 23 studies could not be retrieved, primarily due to being a conference abstract without a corresponding full-text article, 229 full-text articles were assessed for eligibility, and 188 were excluded. As seen in Fig. 1, the primary reasons for exclusion at full-text screening includes: articles being review papers (n = 21); failing to explore factors that influence ADHD diagnosis (n = 40); only reporting on prevalence rates of ADHD in different groups without addressing whether or how these group characteristics relate to the ADHD diagnostic pathway (n = 31); and only investigating community, organisational, systemic and societal level factors (n = 32). For example, Efron et al. [61] investigated predictors of professional service use in children with ADHD, but the factors affecting care did not specifically relate to the ADHD diagnostic process. Another example is Collins et al. [62], who used a population-based survey to examine ethnic disparities in parent-reported diagnosis of ADHD, but its primary aim was to examine trends of ADHD prevalence rates, without addressing how these disparities relate to the ADHD diagnostic process.

Forty-one studies met eligibility criteria and are summarised in Table 1 and in the Supplementary Materials 2. Most studies (26/41) reported the impact of the child-level factors on the overall presence of an ADHD diagnosis [33, 35, 42, 63–85], while a smaller number reported on the specific steps within the ADHD diagnostic process (7/41) [86–92], the age of ADHD diagnosis (4/41) [93–96], or delays within specific steps in the ADHD diagnostic process (4/41) [97–100]. Of the 41 included studies, 17 were from the USA, five from the UK, three from Sweden, two from Germany, two from Italy, two from Taiwan, and the remaining were from Bosnia and Herzegovina, Brazil, Canada, Denmark, Finland, France, India, Ireland, Japan, and Norway. A number of different methodologies were used by included studies, but all were quantitative. None of the included studies reported any conflicts of interest. Most studies were published in peer-reviewed journals, except for three unpublished dissertations [92, 94, 96].

**Table 1:** Included studies and participant characteristics

References	Country	Source	Number of ADHD children/ sample size	Characteristics of ADHD children (age, gender)	ADHD diagnostic process under investigation	Individual factors investigated
Arruda et al. [65]	Brazil	Schools	7114; 505 ADHD-diagnosed children; 277 ADHD-probable children	5–18 years 59.6% 5–9 years; 32.3% 10–13 years; 8.1% 14–18 years 49.9% male	Likelihood of a clinical diagnosis of ADHD and probable-ADHD	Income class School type Gender Ethnicity Population density Age School performance
Arya et al. [97]	India	CAMHS outpatient	57 children with ADHD	6–16 years 85.9% 6–12 years; 14% 13–16 years 8 70.2% male	Delay in contacting specialist services	Gender Age Urbanicity SES Subtype Comorbidities Family type
Bannett et al. [93]	US	Paediatric primary health care network	195 children with ADHD; 29,213 non-ADHD children	2–5 years 72.8% male	The presence and timings of a clinical diagnosis of ADHD	Comorbidities Gender Insurance type
Barry et al. [86]	US	Schools	1044 children with ADHD	Mean age = 8.7 years (SD = 1.6) 66.4% male	Referral and parental recognition of ADHD	Race SES Gender Age ADHD symptoms ODD/CD symptoms Anxiety/ depression symptoms Teacher-rated classroom impairment
Bax et al. [64]	US	Schools	1068 children; 245 ADHD-diagnosed children; 259 ADHD-probable children	5–13 years 51.9% male	The likelihood of a clinical diagnosis of ADHD using the ADHD-probable group only	SES Insurance Race/ethnicity Single parenthood Gender Urbanicity
Bonati et al. [98]	Italy	ADHD reference centres	2262 children; 1553 ADHD-diagnosed children	5–17 years (median age = 9) 85% males	ADHD diagnostic evaluation pathway duration	Age Gender Psychiatric comorbidities Chronic medical comorbidities Clinician-rated ADHD severity
Bonati et al. [65]	Italy	ADHD reference centres	2856 ADHD-diagnosed children	6–17 years (mean age = 9.3 years; SD = 2.5) 86% males	The presence of a clinical diagnosis of ADHD	Relative age
Bussing et al. [87]	US	Schools	389 children with ADHD	Mean age = 7.8 years (SD = 1.8) 52% male	Parental problem recognition, professional evaluation, and clinical diagnosis of ADHD	Gender Age Race SES Insurance Parent-rated severity
Chen et al. [66]	Taiwan	National Health Insurance Database	149,384 ADHD-diagnosed children	3–17 years old 50.8% male	The likelihood of a clinical diagnosis of ADHD	Urbanicity Relative age
Chen et al. [67]	Taiwan	National Health Insurance Database	~ 8714 ADHD-diagnosed children	4–17 years old	The likelihood of a clinical diagnosis of ADHD	Relative age
Coker et al. [35]	US	Schools	4297 children; 8% with clinical levels of ADHD symptoms and 8% diagnosed with ADHD	-Fifth grade at first data collection (median age = 11 years) 51.1% male	The likelihood of the presence of a clinical diagnosis of ADHD	Race/ ethnicity
Dizdarevic et al. [68]	Bosnia and Herzegovina	Schools	1935 children; 97 children with clinical levels of ADHD symptoms; 138 children clinically diagnosed with ADHD	6–15 years (mean age = 10.2; SD = 2.1) Children with completed parent-questionnaires: 45.2% male Clinically-diagnosed ADHD children: 63.8% male	The presence of a clinical diagnosis of ADHD and probable-ADHD	Gender
Elder [69]	US	Early childhood longitudinal study	11,784 children; 6.4% diagnosed with ADHD	Mean primary school starting age = 5.4 years (SD = .4)	The likelihood of a diagnosis of ADHD	Relative age
Evans et al. [70]	US	National Health Interview Survey	35,343 children; 8.6% was diagnosed with ADD/ ADHD	7–17 years (mean age = 11.8) 50.7% male	The likelihood of a clinical diagnosis of ADHD	Relative age
Froehlich et al. [33]	US	The National Health and Nutrition Examination Survey	3082 children; 222 ADHD-probable children	8–15 years 51% male	The likelihood of a clinical diagnosis of ADHD among ADHD-probable children	ADHD subtype Age Gender Race/ ethnicity Income poverty Health insurance status
Halldner et al. [71]	Sweden	National registers	12,233 ADHD-diagnosed children	6–17 years	The likelihood of an ADHD diagnosis	Relative age
Hlavaty [94]	US	ADHD Center for Evaluation and Treatment	1331 ADHD-diagnosed children	3–18 years (mean age = 9.1, SD = 3.6) 70.7% male	Age at ADHD diagnosis	Parent- and teacher-rated ADHD symptoms Parent-rated impairment Parent- and teacher-rated behavioural problems IQ Social functioning Emotional intelligence
Hoang et al. [95]	UK/ England	General Practitioners Research and Surveillance Centre network	3470 children with ADHD	< 19 years old 81.3% female (95% CI = 80.0–82.6%)	Age at ADHD diagnosis	Family size Family structure Urbanicity SES
Huss et al. [72]	Germany	Nationwide research database	667 ADHD-diagnosed children; 644 ADHD-probable children	3–17 years ADHD-diagnosed children: 81.7% male ADHD-probable children: 63.0% male	The likelihood of the presence of a clinical diagnosis of ADHD and probable-ADHD	Gender SES
Karlstad et al. [73]	Norway	National Registry	509,827 children; 17,105 children diagnosed with ADHD by specialists; 16,284 children diagnosed with ADHD by GP	6–14 years Whole sample population: 51.2% male Children with specialist ADHD diagnosis: 72.8% male Children with GP diagnosis of ADHD: 72.4% male	The likelihood of a diagnosis of ADHD	Relative age
Klefsjö et al. [88]	Sweden	Child and Adolescent Psychiatric outpatient	100 ADHD-diagnosed children	17 years 50% male	Referral (referral reasons and source) and diagnostic process (age at first visit, age at first diagnosis, number of visits before receiving diagnosis, time span between first visit and diagnosis)	Gender
Layton et al. [74]	US	Truven Health MarketScan Research Database	71,672 children; 534 ADHD-diagnosed children	4–17 years	The presence of a diagnosis of ADHD	Relative age
Madsen et al. [75]	Denmark	National Birth Cohort	1373 ADHD-diagnosed children; 653 ADHD-probable children with the absence of a registered ADHD diagnosis	Mean age at the end of follow-up = 12.5 years (SD = 1.4) Total sample: 51.2% male ADHD-diagnosed children: 79% male ADHD-probable children but not diagnosed: 67% male	Clinical diagnosis of ADHD vs. ADHD-probable children with an absent of ADHD diagnosis	Gender SES Family status
Morgan et al. [76]	US	Early Childhood Longitudinal Study (ECLS)	6550 children; ~ 150 children with ADHD	Mean age at the 60-month assessment = 64.7 months (SD = 3.8) 61.2% male	The likelihood of an ADHD diagnosis, and being in the lowest 9.2% of ADHD-related behavioural functioning	Race/ethnicity Primary language Gender SES Family structure Cognitive functioning Health insurance coverage
Morgan et al. [77]	US	ECLS	17,100 children; 6.8% children diagnosed with ADHD	Mean age at first data collection = 68.5 months (SD = 4.4) 51.2% male	The likelihood of an ADHD diagnosis	Gender Race/ethnicity Primary language Health insurance Externalising problem behaviour Academic achievements SES
Morgan et al. [42]	US	ECLS	1070 children diagnosed with ADHD	Mean age at first data collection = 68.5 months (SD = 4.4) 51.2% male	The risk of being above-average in various intelligence and behavioural variables prior to ADHD children’s initial ADHD diagnosis (as a proxy of overdiagnosis); and the likelihood of being diagnosed with ADHD among children displaying above-average intelligence and behavioural variables the year prior	Race/ethnicity SES
Morrow et al. [78]	Canada	National health databases	937,943 children	6–12 years (mean age = 7.8) 51.3% male	The presence of an ADHD diagnosis	Relative age
Mowlem et al. [79]	Sweden	Nationwide cohort study	19,804 children; 650 children clinically-diagnosed with ADHD; 2556 ADHD-probable children	9 years at first data collection 50.64% males ~ 2.5:1 male-to-female ratio in clinically-diagnosed children ~ 1.8:1 male-to-female ratio in ADHD-probable children	The predictive association between various clinical characteristics and a clinical ADHD diagnosis, according to gender	Gender
O’Connor and McNicholas [80]	Ireland	National Longitudinal Study	71 ADHD-diagnosed children; 582 ADHD-probable but undiagnosed children; 7915 non-ADHD controls	9 years at first data collection ADHD-diagnosed children: 74.8% male ADHD-probable children: 72.1% male Non-ADHD control group: 49.4% male	ADHD-diagnosed vs. ADHD-probable but undiagnosed group	Gender Single-carer household Social class Parental education Equivalised income Parent- and teacher- rated ADHD symptoms General health Conduct problems Emotional symptoms Peer relationships
Oxley [96]	US	Schools	155 ADHD-diagnosed children	6–11 years (mean age = 9.5) 81.9% male	Age at ADHD diagnosis	Subtype
Purper-Ouakil et al. [99]	France	Child and Adolescent Psychopathology outpatient clinic	129 children with ADHD	6–16 years (mean age = 125.5 months; SD = 34.1) 85.3% male	Diagnostic delay (time between first consultation and definite diagnosis)	Co-morbid internalising and externalising disorders Subtype Gender ADHD severity
Root et al. [81]	UK	UK Clinical Practice Research Datalink	1,039,430 children	4–15 years (median age at study entry was 4.0 years; IQR = 4.0–5.0) Median age at ADHD diagnosis = 8.0 years (IQR = 6.7–9.7) 51.1% male	The likelihood of an ADHD diagnosis	Relative age
Sayal et al. [82]	Finland	National registries	6136 children with ADHD	7–19 years (mean age = 9.4; SD = 2.4) 84.8% male	The likelihood of an ADHD diagnosis	Relative age
Sayal et al. [89]	Great Britain	British Child and Adolescent Mental Health Surveys (B-CAMHS)	176 children with ADHD in the 2004 survey; 238 in the 1999 survey	2004 survey: 5–16 years (mean age = 10.3; SD = 3.2); 84% male 1999 survey: 5–15 years (mean age = 10.0; SD = 2.9); 81% male	Parental recognition and contact with professionals	Comorbid emotional or behavioural conditions Parent- and teacher-rated impairment Gender Age Single parenthood House ownership Symptom severity General health
Sayal et al. [90]	Great Britain	B-CAMHS	232 children with ADHD	5–15 years (mean age = 10.0; SD = 2.9) 81% male	Parental recognition and contact with professionals	Comorbid emotional or behavioural conditions Parent- and teacher-rated impairment Gender Age Single parenthood House ownership Symptom severity General health
Sayal et al. [91]	England	CAMHS and GPs records	127 children with ADHD; 58 ADHD-probable children; 40 children recognised with ADHD by GP	ADHD-probable children: mean age = 8.3 years (SD = 1.7) GP-recognised children: mean = 7.7 years (SD = 1.7)	The likelihood of GP recognition and referral of ADHD in children	Parent-rated hyperactivity symptoms Conduct problems Emotional problems
Schwandt and Wuppermann [83]	Germany	Statutory health insurance records	Roughly 7.2 million children; ~ 3.8% diagnosed with ADHD	4–14 years	The likelihood of an ADHD diagnosis	Relative age
Sikov et al. [84]	US	Primary care paediatric clinics	2,212 children; 264 children diagnosed with ADHD	6–11 years (mean age = 8.6; SD = 1.7) 49.5% male	The likelihood of a clinical ADHD diagnosis	Primary language Psychiatric comorbidities Medical comorbidities Gender Insurance type Age Race/ ethnicity
Staniszewski [92]	US	Schools	115 children with ADHD; 21 children referred to specialist; 94 non-referred ADHD children	Kindergarten to third grade 71.3% male	ADHD students referred to specialist services vs. non-referred ADHD-probable students	Gender Relative age Intelligence Academic Competence Learning problems Adaptability Aggression Conduct problems
Stevens et al. [85]	US	Medical records	~ 26,450 children; 902 children diagnosed with ADHD	3–18 years	The likelihood of an ADHD diagnosis	Race/ ethnicity Age Insurance type
Yamauchi et al. [100]	Japan	Specialised child psychiatric services	387 children with ADHD	Mean age = 11.2 years (SD = 3.0) 88.3% male	Time between initial parental concern and first visit to child psychiatric services	Age Household income Behavioural problems Commute time Parental education Gender Parent-rated impairment Developmental delay

Critical appraisals of all included studies can be found in the Supplementary Materials 3. The quality appraisal revealed that the highest proportion of included papers (34; 82.9%) received low ratings for the domains of participant selection and measurement of child-level factors. This is due to the high number of population-based cohort studies and large community-based samples, which have a relatively low risk of selection bias. For child-level factors, much of the data relied on trustworthy sources such as medical records, and/or used well-validated measures as the Strengths and Difficulties questionnaire (SDQ) [101]. Conversely, a significant proportion of studies (12; 29.3%) had a high risk of bias caused by inadequate blinding of outcome assessments. Moreover, 12 studies (29.3%) inadequately considered potential confounds, a concerning observation given that the receipt and timing of ADHD diagnosis depends on a complex interplay between child and contextual factors. Additionally, a significant number of studies (20; 48.8%) did not report their approach to handling missing data.

Overall, 16 child-level factors were identified as influencing the diagnosis of ADHD in children and adolescents. The clinical factors include ADHD subtype, comorbid mental disorders, behavior problems, internalizing symptoms, ADHD symptom severity, impairment, social and cognitive functioning, and general health; whereas the sociodemographic factors included age, gender, relative age, race/ethnicity, SES, insurance status, urban or rural residence and family structure.

#### Subtype

Four papers examined the impact of ADHD subtypes on the receipt or timing of an ADHD diagnosis [33, 96, 97, 99]. In a nationally-representative sample of US children [33], children who met DSM-IV criteria for ADHD-C were approximately 10% more likely to have been previously diagnosed with ADHD than children who met criteria for ADHD-HI only. A further 10% of children who met criteria for ADHD-C were more likely to have been previously diagnosed with ADHD compared to those who met criteria for ADHD-I only. However, these differences were not statistically significant.

Regarding age at diagnosis, in a student sample from rural northeast Georgia, children with ADHD-C were previously identified and diagnosed by a psychologist or psychiatrist at a significantly younger age than their ADHD-I counterparts [96]. Children with ADHD-C demonstrated the fastest access to specialist services for evaluation from the onset of symptoms, followed by ADHD-HI, while children with ADHD-I experienced the most substantial delay in referral to a specialist [97]. Children with ADHD-I had a median delay of 6 years, which was twice as long as the delay observed in ADHD-C children. However, another study showed that once in contact with professionals, there were no significant differences in the time required for a formal diagnosis among children with varying ADHD subtypes [99]. Overall, findings suggest that children with the ADHD-I subtype may experience the most delayed diagnosis, and that the ADHD subtype may exert a more pronounced influence on the diagnostic steps preceding specialist services, rather than on the diagnostic process once the child is in contact with specialist services.

#### Mental health comorbidities

Six papers examined the effects of diagnoses of comorbid mental disorders, irrespective of the exact nature of these disorders, on ADHD diagnosis [84, 89, 90, 93, 97, 98]. These studies grouped various mental health conditions together as one factor. For instance, Arya et al. [97] combined ODD, CD, and substance use, whereas Sikov et al. [84] combined anxiety, depression, disruptive behavior disorders, learning and developmental disorders, and mood disorders together as one group. Findings regarding the impact and directionality of mental health comorbidities on the identification of ADHD were inconsistent. Sikov et al. [84] suggested that the presence of comorbid disorders increased the likelihood of receiving an ICD-10 ADHD code in primary care health records. However, while a population-representative sample from the 2004 British survey indicated that the presence of comorbid mental disorders increased parental recognition, professional contact, and referral [89] these factors were not significant predictors in the 1999 survey conducted five years earlier [90]. As both studies employed the same study design and methodology, the different results may be attributed to changes in context between the study years.

Regarding the timing of diagnosis, two studies suggested that the presence of comorbid disorders may reduce the duration of the diagnostic process, showing that children with comorbid conditions had a decreased delay in help-seeking and contacting specialist services in a newly registered outpatient sample [97], and a younger age at recorded clinical ADHD diagnosis in primary care health records [93]. However, Bonati et al. [98] found that comorbidity did not significantly affect the time taken to receive a diagnostic evaluation for children who had already enrolled into regional specialised ADHD referral centres.

These studies all originated from diverse countries, encompassing the USA [84, 93], UK [89, 90], Italy [98] and India [97]. There was also study variation in the types of comorbid diagnoses examined. For instance, Arya et al. [97] focused on ODD, CD, and substance use, whereas Sikov et al. [84] included a broader range of diagnoses, such as anxiety, depression, disruptive behavior disorders, autism spectrum and developmental disorders, and mood disorders. Given these disparities, and the heterogeneity in findings, a definitive conclusion cannot be drawn.

#### Disruptive behavior problems

Disruptive behavior problems, including conduct problems, aggression, and ODD/CD symptoms, were examined in eight studies [77, 80, 86, 91, 92, 94, 99, 100]. In these studies, disruptive behavior was assessed via parent-report [80, 99, 100], teacher-report [77, 86, 92], or by both informants [91, 94] using well-established instruments or semi-structured interviews.

Among these papers, three studies indicated that disruptive behavior facilitated the diagnosis of ADHD. Conduct problems and aggression were linked to an increased likelihood of an ADHD diagnosis [77], recognition and referral by teachers [92], and PCPs [91], likely due to enhanced impairment and problem recognition. The potential confounding variables adjusted by these studies included age, race/ethnicity, SES, and academic achievement, except for Staniszewski [92], which did not include any adjusted models. However, two studies did not find a relationship between disruptive behavior and ADHD diagnosis. Barry et al. [86] found that disruptive behavior had no significant effect on the likelihood of parental recognition and acceptance of the recommendation for an ADHD referral. O’Connor and McNicholas [80] also did not find a significant difference between the behavior problems of children diagnosed with ADHD and those exhibiting ADHD symptoms at age nine who were not diagnosed at the time, despite diagnosed 9-year-olds tending to score higher on behavior problems than their undiagnosed peers. The authors controlled for additional potential confounding variables, such as ADHD symptoms, cognitive ability, general health and service engagement, which may have moderated the relationship between behavior problems and ADHD diagnosis.

Three studies examined whether disruptive behavior problems affected the timing of ADHD diagnosis, all with differing findings. Hlavaty [94] found that the severity of disruptive behavior, difficult temperament and non-compliance at home and school contributed to an earlier age of ADHD diagnosis. Purper-Quakil et al. [99] found no association between the presence of externalizing disorders and the time taken to receive a formal diagnosis once the child reached specialist services for evaluation. In contrast, Yamauchi et al. [100] found that parent-rated behavior problems were significantly related to a longer time lag between initial parental concern and the first visit to child psychiatric services. This study was conducted in Japan, representing the only non-Western study among the eight reviewed. In summary, findings regarding the influence of disruptive behavior on ADHD diagnosis were varied across the studies, leaning towards comorbidity being a facilitator of diagnosis. These discrepancies may be attributed to variations in study methodology and potential cultural influences.

#### Internalizing symptoms

Four studies investigated the influence of internalizing symptoms on the timing and receipt of an ADHD diagnosis [80, 86, 91, 99], defined in this review as anxiety and depression symptoms, reported by parents [80, 91, 99] and teachers [86, 91]. These studies showed that internalizing symptoms were generally not associated with ADHD diagnosis. The presence of internalizing symptoms did not affect parents [86] or PCPs’ [91] problem recognition and decision to refer children for a professional evaluation, or whether or not children who exhibited diagnosable ADHD symptoms were diagnosed with ADHD at age nine [80]. However, Purper-Quakil et al. [99] found that internalizing problems led to a significantly longer diagnostic process after contact with specialist services. Overall, internalizing problems may increase the time taken for health professionals to evaluate a child’s ADHD symptoms, but their presence does not appear to affect problem recognition, referral or the presence of ADHD diagnosis more generally.

#### ADHD symptom severity

Nine studies within this review explored the impact of ADHD symptom severity on diagnosis [80, 86, 87, 89–91, 94, 98, 99]. Findings varied, with discrepancies potentially due to the use of different measures and informants. Some studies relied on parent report [91, 99], teacher report [80, 86], both parent and teacher report [87, 94], while others used clinician-based assessments [89, 90, 98]. Different informants may observe child behavior in different contexts or apply different thresholds for whether different symptoms are considered severe or impairing. For example, O’Connor and McNicholas [80] found significantly higher parent-rated hyperactivity/inattention for children diagnosed with ADHD compared to undiagnosed children with probable ADHD, while no such distinctions emerged with teacher ratings.

In terms of parent-rated ADHD symptoms, hyperactivity and impulsivity symptoms facilitated recognition [87], formal diagnosis [80, 87], and the early identification of ADHD [94]. However, these symptoms did not affect the time taken by clinicians to make a diagnosis [99], nor whether PCPs recognised a problem in the child with ADHD [91]. No association was found between teacher-rated symptom severity and the likelihood of recognition [86, 87], referral [86], clinical evaluation [87], or formal diagnosis [80, 87]. The only significant outcome for teacher-rated symptoms was in Hlavaty’s [94] study, where greater teacher-reported inattentive and hyperactivity symptoms was associated with a younger age of ADHD diagnosis in children referred to an ADHD centre for evaluation. When symptom severity was assessed by clinicians, higher severity was associated with increased past-year communication with specialist health services [89, 90], and a shorter ADHD diagnostic evaluation period [98]. Nevertheless, clinician severity ratings were not related to parental problem recognition after adjusting for covariates [89, 90]. Overall, the influence of ADHD symptom severity differed across informant at different stages of the diagnostic process, emphasizing the need to consult with multiple informants at each step of the diagnostic process.

#### Impairment

Impairment denotes significant difficulties faced by the child across various life domains that causes diminished well-being. Most studies (3/4), with the exception of Yamauchi et al. [100], suggested that better well-being may predict a delayed ADHD diagnosis in certain contexts. Using a population-based sample from the UK, lower impairment was related to reduced parental recognition and willingness to refer their child [90], and decreased contact with professionals [89, 90]. Sayal et al. [89] also found that less impairment was significantly associated with reduced parental recognition in their initial bivariate analysis, similar to the 2006 study. However, this association did not persist after controlling for other variables, including house ownership, ADHD severity, and the presence of a comorbid emotional or behavioral disorder. Similarly, children with better quality of life demonstrated a significantly older age of ADHD diagnosis [94].

In contrast, Yamauchi et al. [100] found that impairment was not significantly related to a delay in accessing child mental health services in Japan. However, as mentioned in the above section (see ‘Disruptive Behavior Problems’), Yamauchi et al. [100] was the only study conducted in Asia. As the authors did not find a significant effect of impairment in family or school life on the time lag between initial parental concerns and first child mental health service access, they decided not to proceed with a more complex model accounting for other factors such as child’s age, family structure, area of residence and SES in their analysis. In contrast, all other studies were carried out in English-speaking Western countries such as the UK [89, 90] or the USA [94], and these studies accounted for potential confounds in their analyses. Hence, cultural differences and other mediating factors may contribute to this conflicting finding. SES, ADHD and comorbid symptom severity may affect the relationship between impairment and ADHD diagnosis. The use of parents as the informant for ADHD-related impairment may also influence this relationship. Sayal et al. [90] found that HKD criteria, used as a proxy for symptom severity, was not linked to parental recognition, acceptance of referral, or contact with specialist services, but parent-reported impairment and strain to family were related to these outcomes, underscoring the influential role of parental perceptions. Some additional potential confounding variables may not have been controlled for in study analyses, possibly leading to an overestimation or underestimation of the association between ADHD symptom severity and recognition, referral and service contact.

#### Social and cognitive functioning

The influence of child social and cognitive functioning on ADHD diagnosis was explored in eight studies [63, 76, 77, 80, 86, 92, 94, 100]. Study findings were mixed, seemingly contingent on the specific conceptualization of these factors or the measurement tool employed to assess social or cognitive functioning. Interestingly, two studies employing standardised age-appropriate psychometric tests to directly assess cognitive functioning found no significant relationship between this factor and the presence [76], or age of ADHD diagnosis [94]. However, when cognitive ability was measured using educational achievement, performance and progress as proxies, results were more varied. Enhanced academic achievement, positive learning-related behavior, and higher verbal and numerical reasoning skills were associated with a reduced likelihood of ADHD diagnosis [63, 77], even when only children with confirmed clinical levels of ADHD symptoms were included in the study [80]. However, teacher-rated school performance did not significantly influence parental recognition or the decision to refer children to professional services [86]. Staniszewski [92] reported mixed findings, noting that adaptability, which was considered a proxy for intelligence, was significantly lower in referred children compared to non-referred ADHD-probable children, while child IQ and academic competence ratings were not significantly related. The study’s limitations, such as a small sample size, the dichotomous categorisation of intelligence and academic competence as above or below average, and the sole reliance on teacher report may have contributed to their mixed results. It is crucial to consider the diversity of informants and measures, recognising that school performance, especially when solely evaluated by teachers, may be strongly influenced by children’s behavior. However, findings suggest that performance on standardized assessments of cognitive functioning does not appear to broadly influence the receipt and timing of an ADHD diagnosis.

Regarding other measures of social and cognitive development, Yamauchi et al. [100] found that developmental delay did not affect the time between first parental concern and the child’s first visit to specialist services. Conversely, better social functioning delayed the identification of ADHD. O’Connor and McNicholas [80] revealed that children with better peer relationships were less likely to receive an ADHD diagnosis at age nine, while Hlavaty [94] found that children with better social functioning and higher emotional intelligence were diagnosed later. While some study findings suggested cognitive ability or academic performance and social functioning can prevent or delay an ADHD diagnosis, these factors, especially when reported by informants rather than based on standardized tests, may be susceptible to confounding variables, such as informant-child relationships, externalizing symptoms, and informant knowledge and biases.

#### Physical health

Five studies investigated the impact of child physical health on ADHD diagnosis [80, 84, 89, 90, 98]. Overall, it appears that poor health and the presence of a comorbid medical condition can facilitate ADHD diagnosis [80, 84, 98]. Using a representative population sample, O’Connor and McNicholas [80] observed that children diagnosed with ADHD by age nine exhibited poorer physical health than those with clinical levels of ADHD symptoms who remained undiagnosed. Sikov et al. [84] reported that in a hospital-based primary care paediatric clinic of school age children, those with an asthma diagnosis had significantly higher odds of an ADHD diagnosis, even after adjusting for symptoms of inattention. Bonati et al. [98] found that among children who were referred to specialised ADHD centres with suspected ADHD, those with comorbid chronic medical conditions had a shorter waiting time before receiving a formal ADHD diagnosis. These findings may reflect greater severity of symptoms and/or functional impairment from the presence of both conditions [80, 84], or because children with chronic health conditions are more connected to health services, facilitating detection and treatment access [84].

Results from some studies were non-significant when examining specific diagnostic steps or particular health problems. For example, an obesity diagnosis was not associated with an increased odds of an ADHD diagnosis [84]. Child health was also unrelated to parental problem recognition [89, 90]. Whether child health was associated with a higher likelihood of specialist contact varied, with Sayal et al. [90] reporting a positive link, while a study conducted five years later found no effect [89].

#### Gender

Twenty-two studies examined gender in relation to ADHD identification. Boys were consistently more likely to be diagnosed with ADHD [33, 63, 68, 72, 75–77, 79, 84, 87], or to be diagnosed at an earlier age than girls [88], with only two exceptions where no gender effects were present [64, 80]. These gender disparities have been observed across cultures, including the US [33, 76, 77, 84, 87], Brazil [63], Bosnia and Herzegovina [68], Germany [72], Sweden [79, 88], and Denmark [75].

Studies that examined the influence of gender on specific filters within the diagnostic process did not produce any significant findings. Child gender did not influence parental problem recognition [86, 87, 89, 90], their decision to refer the child or not when recommended [86], whether they had contact with professional services [89, 90, 92], or the time between initial recognition and the first visit about concerns related to ADHD [97, 100]. Similarly, gender did not impact the duration of the ADHD diagnostic evaluation [98, 99]. Each specific step within the diagnostic process was only examined by a few studies, and not all steps have been investigated simultaneously within each study, limiting the conclusions that can be drawn.

The relationship between gender and ADHD diagnosis may also be moderated by other factors. For instance, Bax et al. [64] found that male gender predicted the receipt of an ADHD diagnosis generally, but this gender bias disappeared when only considering ADHD-probable children and controlling for other variables, such as income, primary caregiver educational level, presence of health insurance, race/ethnicity, school district, and single parenthood. Bannett et al. [93] found that compared to children who received a formal diagnosis of ADHD, children with only a symptom-level documentation of ADHD by PCPs were more likely to be girls. Moreover, Klefsjö et al. [88] found that girls with ADHD were more frequently referred to specialist services for emotional concerns rather than ADHD symptoms, while Mowlem et al. [79] found that externalizing symptoms were more strongly associated with clinically diagnosed ADHD in girls than boys. Taken together, these findings suggest that the link between ADHD diagnosis and gender may stem from a range of systemic factors, such as SES, societal norms and expectations around gender in relation to child behavior, as well as biases in how parents and clinicians perceive ADHD to present in boys versus girls.

#### Age

Eleven studies explored the role of child age, although none specifically focused on age as the primary risk factor [33, 63, 84–87, 89, 90, 97, 98, 100]. A considerable number of studies found that age did not significantly impact ADHD identification, including parental recognition [86, 89, 90], contact with specialist services [89, 90, 97], or a formal diagnosis [33, 63, 87]. In contrast, a study conducted in the US found that children older than 7 years were twice as likely to have been diagnosed with ADHD compared to younger children [84]. This study defined ADHD diagnosis as the presence of a lifetime diagnosis, therefore, this finding may be due to children with ADHD symptoms being diagnosed cumulatively over time, rather than an inherent bias based on age. Nevertheless, Stevens et al. [85] indicated that an ADHD diagnosis was most likely to be recorded in the medical records of 7–12-year-old children in the US, three times more likely than for 3–6-year-olds and 13–18-year-olds.

When considering the speed of the ADHD diagnostic process, Yamauchi et al. [100] found that younger children experienced a longer delay in accessing specialist services for evaluation, whereas Bonati et al. [98] indicated a shorter diagnostic evaluation process for younger children. However, these two studies differed in temporal scopes; Yamauchi et al. [100] concentrated on the interval from symptom onset to the initial visit to child psychiatric services, while Bonati et al. [98] focused on the duration from the initial request to the ADHD centre to the moment of diagnosis, limiting the conclusions that can be drawn.

#### Relative age

Twelve studies examined the influence of children’s ages relative to their school grade on their likelihood of being diagnosed with ADHD in various countries, including the USA [69, 70, 74]; Taiwan [66, 67]; Italy [65]; Sweden [71]; Norway [73], Canada [78], Finland [82]; UK [81]; and Germany [83]. These studies employed diverse study designs and methods. For instance, different sources were used to ascertain ADHD diagnosis, such as medical records [66, 67, 71, 73, 78, 81–83], parent report [69, 70], and insurance claim records [74]. They were also conducted in nations with different school-starting month and birth date cut-offs for eligibility for school entry, i.e., January [65, 71, 73, 78, 82], September [66, 67, 70, 74], or different birthday cut-offs within their own study [69, 81, 83]. Despite these methodological differences, all 12 studies consistently found that relatively younger children within their school year were more likely to receive a formal ADHD diagnosis.

Studies that stratified the analysis with other child-related factors, revealed that the relative age effect may be influenced by additional factors, including the child’s actual age [65, 67, 74, 81–83], gender [65, 74, 82, 83], ADHD subtype [65], and birth cohort [70, 82]. For example, studies have indicated that the impact of relative age on ADHD diagnosis is strongest in primary school-aged children, with no relative age effect observed in preschool-aged children [74, 83], and the effect in school-aged students appeared to decline or become non-significant with increased age [65, 67, 71, 82].

A popular explanation for the relative age effect is the immaturity hypothesis [e.g., 65, suggesting that younger children may exhibit less maturity in terms of self-regulation, increasing the likelihood that teachers will raise concerns with parents or refer the child to specialist services. Consistent with this hypothesis, Elder [69] discovered that being younger in school is significantly associated with an increased likelihood of being rated by teachers as having clinical levels of ADHD symptoms. This relative age effect is much stronger when derived from teacher rather than parent ratings, underscoring the heightened influence of relative age on teacher perceptions. This is presumed to arise from teachers forming their opinions of a child by comparing them to their peers within the same classroom, better situating them to assess child symptoms and behavior relative to developmental stage. In contrast, Staniszewski [92] examined the relationship between relative age and the likelihood of children with a probable diagnosis of ADHD being referred to professional services and found no significant association. This non-significant finding may be due to methodological differences. Relative age was defined dichotomously (early versus late birthdates, using March 1st and September 1st as cutoffs), limiting the sensitivity of the measure of relative age. Assessing smaller segments within the year and conducting sensitivity analyses may help to detect more nuanced differences, as demonstrated in other studies.

#### Race/ethnicity

Eleven studies investigated the role of race/ethnicity in relation to an ADHD diagnosis, with one study carried out in Brazil [63], and the remaining studies in the USA [33, 35, 42, 64, 76, 77, 84–87]. The Brazilian study found no significant association between child ethnicity and the likelihood of being diagnosed with ADHD. In contrast, studies in the USA tended to show racial/ethnic disparities. Most studies revealed that White children were more likely to receive a prior formal ADHD diagnosis [35, 42, 64, 76, 77, 85, 87], with the exception of two studies [33, 84]. Specifically, Black children were less likely to be diagnosed with ADHD compared to White children, even when presenting with similar symptoms [35, 64, 76, 77, 87]. Similar patterns were observed for Latinx children [35, 77, 85], although two studies did not find a significant effect [64, 76]. Morgan et al. [76] initially observed underdiagnosis of ADHD in Latinx children, but the disparity became statistically insignificant after controlling for the primary language spoken at home. Thus English as a secondary language may as a barrier to awareness, access and communication with professionals in nations where English is the official language. Studies consistently found that children who primarily spoke English had significantly higher odds of receiving an ADHD diagnosis than children whose primary language was not English [76, 77, 84].

It is essential to highlight that while most studies that found race/ethnic disparities in ADHD diagnosis accounted for various potential confounds, most did not control for ADHD symptoms. Sikov et al. [84] however, controlled for inattention and found no differences in ADHD diagnosis between White children and children of other ethnicities. This suggests that the apparent disadvantage for children from minoritized ethnicities in ADHD diagnosis may be influenced by differences in ADHD symptoms, or to the potential over-diagnosis of White children. Morgan et al. [42] attempted to investigate the causes of this disparity by using above-average levels of various intelligence and behavioral variables the year prior to children’s initial ADHD diagnosis as proxies for overdiagnosis. Significantly more White children displayed above-average behavioral, academic, or executive functioning the year before their initial diagnoses compared to children from minoritized ethnicities, suggesting that White children are more likely to be over-diagnosed with ADHD. Only two studies examined the impact of race/ethnicity on different stages of the diagnostic process separately. These studies found that race/ethnicity was not significantly associated with parental recognition of ADHD symptoms [86, 87]. However, Black children were significantly less likely to undergo an ADHD clinical evaluation compared to White children [87].

#### SES

Findings on the relationship between children’s SES and the identification of ADHD were inconsistent across 16 identified studies [33, 42, 63, 64, 72, 75–77, 80, 86, 87, 89, 90, 95, 97, 100]. A considerable number reported no significant association [e.g., 33, 64, 80, while others identified low SES as a barrier [e.g., 63, 72, 75, 97, or a facilitator of an ADHD diagnosis [e.g., 100.

Several factors may contribute to these discrepant results. First, studies were conducted in different countries, with six from the USA, two in the UK, and one each from India, Brazil, Taiwan, Germany, Denmark and Ireland. Notably, the study that found no effect of SES was conducted in Japan [100], highlighting potential cultural influences, such as differences in healthcare accessibility and cost. Bussing et al. [87] noted that low SES families were more likely to report financial barriers to accessing ADHD services than families of higher SES in the USA, where the cost of healthcare is relatively expensive and not universally accessible. Different measures of SES, including income class [63], eligibility for free lunch status [86], occupational class and parental education [80], were also employed to represent SES, thus potentially capturing different constructs with different underlying individual and contextual influences. For instance, Arruda et al. [63] found that income class and school type significantly predicted ADHD diagnosis. However, parental education did not yield the same effect, emphasizing the potential impact of differences in the conceptualization and assessment of SES. Yamauchi et al. [100] further highlighted this issue by demonstrating that higher levels of maternal education predicted a shorter diagnostic process, whereas paternal education showed no significant association, and annual income showed the opposite effect. Thus, while parental education is often used as a proxy for SES, it may have differing effects to income in certain cases and may be more closely linked to caregiver’s recognition of problems, representing a more relational-level factor influencing ADHD diagnosis.

Different interpretations have been provided for the finding that low SES acts as a barrier to ADHD diagnosis. Some authors posited that low SES acts as barrier to the recognition and early identification of ADHD [e.g., 86, 97, some suggested that children from high SES backgrounds are over-diagnosed [e.g., 42, while others acknowledged the possibility of both phenomena [e.g., 63. There is a paucity of studies attempting to distinguish between overdiagnosis and underdiagnosis based on SES. Overall, while most of the included papers showed no significant effects of SES on ADHD identification, inconsistencies in study design, settings, and definitions of SES limit the validity and generalizability of the findings, making it difficult to reach a firm conclusion.

#### Insurance

All eight papers that examined the role of insurance on ADHD diagnosis were conducted exclusively in the USA. Four studies examined the likelihood of an ADHD diagnosis in relation to health insurance [33, 76, 77, 87]. Generally, having health insurance was associated with a greater likelihood of a formal ADHD diagnosis [33, 77, 87]. However, this facilitative effect was more pronounced for older children, as no such effect was observed in children prior to kindergarten entry [76], in contrast to older children [77]. The presence of health insurance also did not affect parental problem recognition [87].

Four studies explored the likelihood of an ADHD diagnosis based on type of insurance [64, 84, 85, 93]. Bax et al. [64] observed a strong positive association between Medicaid receipt and a formal ADHD diagnosis compared to other types of insurance in children with clinical levels of ADHD symptoms. Similar findings were reported by Stevens et al. [85]. Bannett et al. [93] found a higher likelihood of an ADHD diagnosis for children with public or military insurance compared to those with private insurance. However, after adjusting for covariates such as age, sex and inattention symptoms, this effect was not replicated [84], highlighting the importance of considering potential confounds to better understand the relationship between insurance type and ADHD diagnosis.

#### Urbanicity

Six studies explored the relationship between residential location and the diagnosis of ADHD [63, 64, 66, 95, 97, 100], all of which were conducted in different countries. Residing in an urban environment was associated with a decreased likelihood of children receiving a diagnosis of ADHD in Taiwan [66]. Conversely, no impact of urban versus rural residency on diagnosis was observed in the USA [64], or in Brazil [63]. In terms of the speed of the ADHD diagnostic process, Hoang et al. [95] found no significant relationship between the urbanicity of individual’s registered GP surgery and the age at ADHD diagnosis in the UK. However, Arya et al. [97] and Yamauchi et al. [100] found a longer delay in contacting a specialist for ADHD evaluation among children residing in rural areas or in locations with extended commute times to the nearest hospitals in India and Japan, respectively. These studies suggest that urban versus rural residency may impact the timing or receipt of ADHD diagnosis in some nations. Despite mixed findings, a discernible pattern emerges in Asian countries, where residing in areas with lower population density may lead to a delay in seeking specialized ADHD evaluation.

#### Family structure

Six studies explored the effect of family structure on ADHD diagnosis [64, 75, 89, 90, 95, 97]. The prevailing consensus across studies indicated that being raised by a single or double parent had no significant effect on problem recognition, eventual diagnosis, or the age at which the diagnosis was established when accounting for other covariates [75, 89, 90, 95]. Nonetheless, Bax et al. [64] identified that belonging to a two-parent family increased the likelihood of a formal ADHD diagnosis among children with clinical levels of ADHD symptoms. Additionally, Arya et al. [97] found that children in two-parent families experienced a shorter delay in contacting specialists than those in joint families, where three to four generations often live together in the same household, and caregiving responsibilities may extend to grandparents or other relatives. This suggests that family structure may influence the diagnostic process, with two-parent families potentially facilitating faster access to specialists, or conversely, children belonging to a single-parent or joint family household may face unique challenges that impact the timing of an ADHD diagnosis.

## Discussion

This systematic review identified 16 child-related factors associated with the diagnosis of ADHD, namely: ADHD subtype, ADHD symptom severity, level of impairment, comorbid mental disorders, disruptive behavior, internalizing symptoms, social and cognitive functioning, physical health, gender, age, relative age, race/ethnicity, SES, insurance coverage, urbanicity of residence and family structure. As expected, findings indicated that greater impairment may aid recognition and actions conducive to the timely diagnosis of ADHD. Similarly, elevated ADHD symptoms played a facilitative role in ADHD diagnosis. This pattern aligns with the intuitive notion that heightened symptomology translates to increased impairment, thereby expediting recognition and progress along the diagnostic pathway [102]. This facilitation is also evident for ADHD diagnosis in children with more severe externalizing symptoms and poorer social functioning, which is likely attributable to the cumulative impact of these comorbid symptoms alongside ADHD, intensifying distress and impairment [7]. These cumulative difficulties may become increasingly problematic and noticeable to caregivers, teachers, and clinicians, motivating them to actively seek assistance [103, 104]. Current findings also indicated that a diagnosis of ADHD-C, and to a lesser extent ADHD-HI, tended to accelerate the diagnostic process, particularly the steps preceding contact with specialist services. This trend may relate to other clinical factors such as ADHD symptoms, disruptive behavior problems, social and cognitive functioning, and impairment. The impulsive-hyperactive symptoms in children with ADHD-C and ADHD-HI often interfere with social functioning and may therefore draw attention from parents and teachers much earlier [105]. For instance, a vignette study by Moldavsky et al. [106] revealed that children with ADHD-I were deemed to be less impaired and received the least referrals than those with more pronounced hyperactive-impulsive symptoms. Greater concern in relation to child functioning appears to amplify the visibility of ADHD, prompting faster problem recognition, referral, and subsequent diagnosis.

Nonetheless, not all studies found that ADHD symptom severity, disruptive behavior and impairment are not always significantly related to enhanced ADHD diagnosis. These inconsistent findings may be attributable to the differences in study methodology, context, cross-cultural variation, and/or the number and type of confounding variables controlled for in analyses. Potential mediating factors, such as child cognitive ability, health, service engagement, attitudes towards help-seeking, and SES may have influenced study results. Current review findings suggest that the specific relationship between a child’s presentation and an ADHD diagnosis often varies across different stages of the diagnostic process, and these effects may be contingent on the informants involved. Parent and teacher ratings of severity and impairment may reflect preconceived expectations regarding child behavior, or the strain they experience in relation to child behavior [107, 108]. For instance, Gadow et al. [109] found that teacher-rated intelligence and academic performance may be biased, with teacher report reflecting a child’s classroom behavior rather than their cognitive ability. Parent and teacher perceptions of ADHD may be interpreted in the context of their daily experience, concerns, and understanding of ADHD, thus their report may reflect their own distress levels, a strained relationship, or comorbid difficulties rather than the disorder itself [109].

The significance of different informants may also vary at different stages of the diagnostic process. Parent-reported symptom severity appears to influence stages where parents have greater involvement, such as early problem identification [87], but not the time taken by clinicians to make a diagnosis [99]. Sayal et al. [90] found that HKD criteria, used as a proxy for symptom severity, was not linked to parental recognition, acceptance of referral, or contact with specialists. In contrast, parent-reported impairment and family strain was related, underscoring the influential role of parental perceptions in the early stages of the diagnostic process. However, Lecendreux et al. [110] demonstrated that subthreshold ADHD symptoms in early childhood predicted an ADHD diagnosis in adolescence, and children with subthreshold symptoms face heightened risk for later adverse outcomes [111]. These findings emphasize the need for early detection and support for children who show subclinical levels of ADHD symptoms.

Cultural context also plays an important role. A study conducted in Japan [100] produced conflicting findings compared to other included studies conducted in Western countries, particularly regarding the effects of child impairment and disruptive behavior. These discrepancies potentially indicate cultural differences in parental attitudes and behaviors related to seeking help for child behavioral issues [112]. Factors such as stigmatization of mental health problems, reliance on informal social networks, and cross-cultural differences in perceptions of what constitutes normal child behavior, could explain the possible hesitation or reluctance among Japanese parents to actively seek help [113]. Arya et al. [97] investigated reasons for parental delays in seeking help in India and found that common beliefs hindering help-seeking for ADHD included perceiving their child as merely being ‘naughty’, attributing symptoms to normal developmental processes, and viewing behavior problems as stemming from a lack of strict parenting. In contrast, studies in Western countries such as Denmark and the USA, suggested that parents often cite more systemic barriers and healthcare-related stigma, including insufficient information about where to seek help, financial impediments, and negative expectations regarding professional responsiveness or willingness to initiate interventions or provide referrals to services [87, 114].

The role of comorbid mental disorders also produced mixed results. Different types of comorbid symptoms and conditions can differentially affect ADHD diagnosis and they also appear to be sensitive to contextual factors. Internalizing symptoms and disruptive behavior problems may complicate the diagnostic process by overshadowing ADHD symptoms [115]. For instance, the mood instability, inner tension, racing thoughts, and internal restlessness stemming from ADHD might be misconstrued as symptoms of mood or anxiety disorders [115, 116], which results in ADHD-related difficulties being overlooked. On the other hand, symptoms of comorbid mental health problems may act as facilitators, as the opportunity for clinical evaluation plays a pivotal role in the ADHD diagnostic process, regardless of the initial reason for assessment or contact with specialists. This is demonstrated in Barry et al.’s [86] study, where the recognition and referral of ADHD symptoms was influenced by whether children were already in receipt of services but not the severity of symptoms of comorbid disorders. Thus, the impact of comorbid mental health problems on the diagnostic process may either hinder or facilitate a diagnosis, depending on factors such as pre-existing service contact, and the overall presentation of the child, including the degree of overlap with symptoms of comorbid mental disorders.

Unexpectedly, despite ASD being frequently comorbid with ADHD, its relationship with ADHD diagnosis was not explored in the included papers. While Sikov et al.’s [84] study included autism, it was combined with other non-ADHD diagnoses as a factor. They found that children had significantly higher odds of an ADHD diagnosis if they had a comorbid diagnoses of a mental disorder, including ASD, even when inattention scores were adjusted. The presence of ASD symptoms can intensify overall impairment, as individuals experiencing both ADHD and ASD symptoms have been reported to be more severely impaired compared to those with ADHD or ASD alone [117, 118], which can prompt caregivers to seek assistance. Similarly, as discussed earlier, the identification of ASD can trigger a comprehensive clinical evaluation, potentially uncovering any additional challenges during the assessment process and facilitating ADHD diagnosis. On the other hand, given the significant overlap between ASD and ADHD symptoms such as deficits in executive functions and difficulties in social interaction [119, 120], diagnosing one condition over the other can be challenging. Individuals with ASD may also exhibit attention deficits, albeit more dependent on the specific stimuli and intrinsic motivation, complicating differential diagnosis. This symptom overlap can lead to ADHD symptoms being misconstrued as ASD symptoms by clinicians [120]. However, while some studies have investigated these two diagnoses together, they predominantly focus on how ADHD symptoms or diagnosis affect ASD diagnosis rather than the other way round [e.g., 121, 122. Therefore, exploring how the presence of an ASD diagnosis or how ASD symptoms influence the identification or detection of ADHD is an important area for future research.

With regards to sociodemographic factors, our findings indicate that boys had a consistently higher likelihood of receiving an ADHD diagnosis, or being diagnosed at an earlier age than girls, irrespective of clinical severity. This skewed gender ratio has been attributed to potential biases within diagnostic instruments, which may be more aligned with the male phenotypical expression of ADHD [34], leading to an underestimation of symptoms and severity in girls [123]. Alternatively, this gender-biased pattern may be rooted in boys’ greater propensity for exhibiting co-occurring disruptive behavior than girls [124], prompting parents and educators to seek assistance and evaluation [106, 125]. Girls are also more likely to present with the inattentive subtype and internalizing symptoms, which often go unnoticed [126]. The impairment caused by ADHD symptoms, particularly the inattentive subtype, may not become apparent until the later school years when academic demands increase and greater independence is expected from teachers. In contrast, boys may be more readily recognized due to more severe externalizing symptoms, which are deemed more impairing and disruptive in the classroom setting. Research indicates that parents and teachers display gender biases in recognizing problems, seeking help, and making referrals. Mowlem et al. [79] found that externalizing symptoms exhibited a stronger association with clinically diagnosed ADHD in girls than in boys, suggesting that when children’s symptoms deviate from gender role expectations, girls are more likely to garner specialist or parental recognition. Evidence has shown that gender-related disparities also exist in the timing of help-seeking in mental health conditions. Assistance is generally sought more for boys in childhood and early adolescence, while parents and teachers tend to seek help for girls during late adolescence [127], highlighting the complex relationship between gender-related societal norms, biases, and differences in recognising ADHD at different developmental stages.

Concerning race/ethnicity, most studies were conducted in the USA, and revealed that being Black or Latinx was associated with a reduced likelihood of receiving a diagnosis of ADHD than being White. However, there is a notable lack of analysis regarding the relationship between racial/ethnic groups other than Black and Latinx, and ADHD diagnosis in our included study. Specifically, groups such as Asians, Native Americans, and Alaskan Native children were often grouped into an ‘other’ racial/ethnic category, limiting the ability to differentiate between them. A recent study indicated that Asian children also have lower ADHD prevalence rates compared to White children [128], suggesting that racial/ethnic disparities may extend beyond Black and Latinx child populations. Therefore, future studies exploring the impact of race/ethnicity on ADHD identification and diagnosis across a broader range of racial/ethnic groups is warranted.

Moreover, while the evidence for this disparity among Black and Latinx children was consistent, there is a lack of understanding of the specific points in the diagnostic process where race/ethnicity plays a role and the reasons behind it. Several suggestions have been made regarding potential contributing factors. Some families expressed reservations about reporting ADHD symptoms or seeking care due to concerns related to stigma or racism [87]. Healthcare providers may have implicit biases and hold stereotypes against minoritized ethnic groups, potentially influencing their ADHD evaluation process and diagnostic decision-making [129]. Language may form a barrier, especially for Latinx children. Families whose English is not the primary language face challenges in accurately reporting ADHD symptoms to healthcare providers in English-speaking nations [130]. The lack of diagnostic tools in non-English languages further exacerbates this gap in care [131]. Therefore, ensuring culturally relevant care in the families’ preferred language is crucial [132]. Multicultural competency training interventions for clinicians and healthcare providers is essential to mitigate biases and equip them with the necessary awareness, skills and sensitivity in clinical and diagnostic settings [30, 128], and to foster an inclusive culture that encourage help-seeking behavior among parents from diverse backgrounds [133].

Findings for the relationship between SES and the identification of ADHD were inconsistent, potentially due to the diversity of countries in which studies were conducted, as the impact of SES on recognition and help-seeking may be contingent on the healthcare system and cultural norms in each country [127]. In the USA, the high cost of healthcare may result in inadequate ADHD diagnosis in lower SES households due to barriers in accessing healthcare. Barry et al. [86] identified engagement difficulties as a barrier for low SES families, noting challenges in schools reaching out to discuss ADHD symptoms. Bussing et al. [87] also reported that parents from low SES families in the US were more likely to cite financial barriers to accessing ADHD services than families of higher SES. However, in countries with universal healthcare, barriers to access for low SES families may be less pronounced.

Divergent findings relating to SES may also reflect variation in its definition and assessment [134]. While parental education is a common SES indicator, its impact on ADHD diagnosis may differ from other SES variables, as it may be more closely linked to caregivers' perceptions than access to services. Education and knowledge associated with parental education can shape attitudes and beliefs about ADHD, thereby influencing help-seeking behavior [135]. Highly educated parents may possess enhanced communication skills and health literacy, leading to an increased ability to navigate the healthcare system successfully [136]. The type of insurance, which was categorised separately as ‘Insurance’ rather than ‘SES’ in this review, may also serve as a proxy for SES. This categorization decision was driven by the heterogenous use of insurance coverage in the included studies. Some studies considered the presence/absence of insurance, while others compared public, private, commercial, Medicaid, and military insurance. Some studies treated insurance as a distinct variable outside of SES [e.g., 87, while some conceptualized it as an SES factor [e.g., 64. However, most studies showed no significant effects of SES on ADHD identification despite variations in study design, settings, and the conceptualization and assessment of SES.

Although some studies examined the impact of child-level factors as contributing to the over- or under-diagnosis of ADHD, they did not investigate whether these differences primarily stem from underdiagnosis or overdiagnosis. For example, some authors posited that the predictive relationship between race/ethnicity and ADHD diagnosis primarily resulted from the underdiagnosis of Black and Latinx children [e.g., 35, while others attributed it to an overdiagnosis of White children [e.g., 42. Coker et al. [35] attempted to investigate both possibilities, but their focus was on the effects at the medical treatment level rather than identification. Consequently, reports concerning under- and over-diagnosis should be interpreted with caution, and it future studies should use designs that contrast false-positive with false-negative diagnoses to address this research gap [137].

### Strengths and Limitations

There are a number of limitations of the literature included in the current review. There was substantial diversity in study design, definitions and measurement of the predictor and outcome variables, along with the inclusion of covariates in analyses. This diversity prevented the conduct of a meta-analysis, and therefore, the relationship between child-level factors and the receipt and timing of ADHD diagnosis could not be quantified. Moreover, as these factors are context-specific and influenced by cultural norms, the generalizability of study findings to different cultures is limited. Future research in countries beyond the US would provide valuable insights, helping ascertain the extent to which findings pertaining to child factors, such as race/ethnicity and SES, in the US are applicable to diverse cultural contexts. Most studies focused on child-level factors in relation to the likelihood of an ADHD diagnosis as a whole, but the precise juncture at which these predictors influence ADHD diagnosis remains largely unexplored, with only a limited number of studies examining how these factors influenced individual stages of the diagnostic process. The recognition of ADHD symptoms in children and referral to tertiary care centres by adults or non-specialist professionals play pivotal roles in aiding the diagnostic process [91, 99]. Future research should aim to unravel the intricate relationships between sociodemographic variables, such as ethnicity, gender, age and SES, along with their impact on ADHD recognition, referral and evaluation, especially in a non-US context.

There are also several limitations of the current review that should be acknowledged. First, this review is limited to studies that involved children with either a research or formal clinical diagnosis of ADHD. Studies employing vignette-based hypothetical designs to assess the decision-making process regarding the referral or diagnosis of children with different characteristics were not considered. The incorporation of such studies could have provided additional insights on the contextual, cognitive and social factors, and allowing a more in-depth examination of the complexities inherent in the diagnostic process. However, this eligibility criterion did provide greater consistency across studies, and ensured that we focused on real children most in need of early identification and support. Only studies published in English were included, introducing the potential for an underrepresentation of studies from non-English speaking countries. We opted not to employ quality assessment as a criterion for exclusion, aiming to provide a comprehensive overview of the identified factors in the literature. However, this approach carries the potential risk of distorting interpretations if low quality studies are afforded equal weight to high quality studies when interpreting findings. To address this limitation, we presented the quality assessment of all the included papers to highlight the methodological limitations of the included studies.

This review also has considerable strengths. To our knowledge, this is the first review that provides an extensive overview of the broad spectrum of child-related factors influencing ADHD diagnosis, encompassing socio-demographic and clinical factors. It therefore contributes a nuanced, multifaceted understanding of the complexities involved in the various stages of the ADHD diagnostic process. This review captures data from 16 countries, with many included studies drawing from population-based samples, increasing confidence in the validity and generalizability of findings. The incorporation of diverse methodologies, designs and settings, coupled with the use of narrative synthesis, facilitated the integration of heterogeneous findings and captured the context- and culture-dependent nature of ADHD diagnosis. The insights gained from this review also produced clear implications for clinical practice and could inform the development of targeted interventions and policies aimed at minimizing the disparities in ADHD diagnosis in children due to their individual characteristics.

### Conclusion and implications

This review identified ADHD subtype, comorbid mental health problems, ADHD symptom severity, level of impairment, social and cognitive development, physical health, gender, age, relative age, race/ethnicity, SES, insurance coverage, urbanicity of residence, and family structure as child-level factors influencing the receipt and timing of ADHD diagnosis. Meeting criteria for the inattentive subtype, lower functional impairment, being female, belonging to a minoritized ethnic group, and being relatively old for their grade were consistently associated with a delayed or absent of ADHD diagnosis.

This study highlights the crucial role of parental, educator, and clinician perceptions in shaping the diagnosis of ADHD, emphasizing the need for heightened awareness and education around the diverse symptom profiles of ADHD, particularly in groups more likely to be under-diagnosed or to experience diagnostic delays, such as girls, children from minoritized ethnic background, those from lower SES families, and children exhibiting less overt symptoms. Psychoeducation programs aimed at parents, teachers, and PCPs may be particularly beneficial at both the problem recognition and referral stage. Reducing stigma around mental health, especially in cultures such as Japan and India, could also encourage proactive help-seeking behaviors. In contrast, addressing healthcare-related stigma and perceived systemic barriers may be more relevant in Western contexts. At the evaluation stage, ongoing clinician training in the differential diagnosis of childhood mental health conditions and the high rates of comorbidity between ADHD and other conditions, may be critical. Additionally, multicultural competency training for healthcare providers is essential to ensure cultural sensitivity and awareness in clinical and diagnostic settings [30]. To address language and cultural barriers to diagnosis, the development of more culturally sensitive ADHD assessments, including non-English diagnostic tools, is also imperative [132].

Nevertheless, this review highlights the limited research examining how different factors impact each stage of the ADHD diagnostic process, so it is important for future research focus on specific stages of ADHD diagnosis. Incorporating more detailed information on children's comorbid mental health conditions and healthcare utilization, such as referral reasons, and service usage patterns, can help pinpoint critical junctures in the diagnostic process that can promote early ADHD identification. Lastly, further research to explore the intricate relationships among child-related variables, along with their contributions to the underdiagnosis and overdiagnosis of ADHD across different populations is warranted. Addressing these issues can inform and contribute to efforts that improve ADHD identification and mitigate disparities in diagnosis, ensuring timely and appropriate support for children and adolescents with ADHD.

## Supplementary Information

Below is the link to the electronic supplementary material.

Supplementary file1 (DOCX 36 kb)

Supplementary file2 (DOCX 48 kb)

Supplementary file3 (DOCX 35 kb)

Supplementary file4 (DOCX 24 kb)

## Data Availability

No datasets were generated or analysed during the current study.
