# Supplementary material for: Individual child factors affecting the diagnosis of attention deficit hyperactivity disorder (ADHD) in children and adolescents: a systematic review
Source: Eur Child Adolesc Psychiatry. 2024 Oct 7;34(5):1469–96. doi: 10.1007/s00787-024-02590-9 (PMC12122567; doi:10.1007/s00787-024-02590-9)
Supplement: Supplementary file 1 — Supplementary file1 (DOCX 36 kb) [file 787_2024_2590_MOESM1_ESM.docx]

**Supplementary Material 1: Additional Information on Systematic Review Method**

**Table 1**

*A List of Free Search Terms Included in the Search Strategy for the Systematic Review.*

| Keywords/ Concepts | Search terms/ synonyms/ alternative terms |
| --- | --- |
| ADHD | ‘ADHD; ADDH; attention deficit; inattention/ inattentive; hyperactive/ hyperactivity/ hyper-active/ hyper-activity; hyperkinetic. |
| Diagnosis/ Identification | Recognition/recognise; identify/ identification; detection/ detect; diagnosis/ diagnose; referral/ refer; help-seeking; treatment-seeking; misdiagnosis/ misdiagnose; underrecognize; under-diagnosis/ underdiagnose; under-detect/ under-detection. |
| Children | Children/ child; adolescent; youth; teen/ teenager; young people; student. |
| Factors/ Barriers | Barrier; hinder; challenge; promote; facilitate; encourage; determinant/ determinate; predict/ predictor; factor; reason; disparity; issue. |

**Table 2**

*An Overview of Search Methods of Each Database.*

| Records identified in each interface | Record identified in each database | Temporal coverage | Date of search | Limits applied to search |
| --- | --- | --- | --- | --- |
| APA PsycNET  (n = 2615) | PsycINFO (n = 2521)  PsycEXTRA (n = 94) | 1887 to present  1938 to present | 30/01/2023 | Date limits; exclude adult populations, irrelevant document type and methodologies |
| PubMed.gov  (n = 2292) | MEDLINE (n = 2292) | 1946 to present | 30/01/2023 | Exclude adult population and irrelevant publication types. |
| Embase.com  (n = 2980) | Embase (n = 2354)  MEDLINE (n = 1906)  Preprints (n = 4) | 1974 to present  1974 to present  2013 to present | 29/01/2023 | English limits; Exclude adult population and irrelevant publication types. |
| EBSCOhost  (n = 1144) | ERIC (n = 924)  Opendiss (n = 220) | 1966 to present  1933 to present | 29/01/2023 | English limits; date limits |

**Table 3**

*An Example of Search Syntax in MEDLINE, including the Number of Records Found.*

| Search No. | Query | Filters | Results |
| --- | --- | --- | --- |
| #1 | (ADHD[MeSH Major Topic]) OR (ADHD[Title] OR ADDH[Title] OR "attention deficit"[Title] OR inattenti*[Title] OR hyperactiv*[Title] OR "hyper activ*"[Title] OR hyperkine*[Title]) |  | 39,416 |
| #2 | recogni*[Title/Abstract] OR identifi*[Title/Abstract] OR detect*[Title/Abstract] OR diagnos*[Title/Abstract] OR refer*[Title/Abstract] OR "help seek*"[Title/Abstract] OR "treatment seek*"[Title/Abstract] OR misdiagnos*[Title/Abstract] OR underrecogni*[Title/Abstract] OR underdiagnos*[Title/Abstract] OR underdetect*[Title/Abstract] |  | 8,536,079 |
| #3 | child*[Title/Abstract] OR adolescen*[Title/Abstract] OR youth*[Title/Abstract] OR teen*[Title/Abstract] OR young[Title/Abstract] OR student*[Title/Abstract] |  | 2,552,510 |
| #4 | barrier*[Title/Abstract] OR hinder*[Title/Abstract] OR challeng*[Title/Abstract] OR promot*[Title/Abstract] OR facilitat*[Title/Abstract] OR encourag*[Title/Abstract] OR determina*[Title/Abstract] OR predict*[Title/Abstract] OR factor*[Title/Abstract] OR reason*[Title/Abstract] OR disparit*[Title/Abstract] OR issue*[Title/Abstract] |  | 9,324,358 |
| #5 | (endocrin*[Title/Abstract] OR cardi*[Title/Abstract] OR heart[Title/Abstract] OR thyroid*[Title/Abstract] OR urin*[Title/Abstract]) OR (College[Title] OR universit*[Title] OR undergraduate[Title] OR "higher education"[Title] OR postsecondary[Title] OR adult*[Title]) |  | 3,287,326 |
| #6 | #1 AND #2 AND #3 AND #4 |  | 4,653 |
| #7 | #6 NOT #5 |  | 3,999 |
| #8 | #6 NOT #5 | Books and Documents | 8 |
| #9 | #6 NOT #5 | Books and Documents, Case Reports | 63 |
| #10 | #6 NOT #5 | Books and Documents, Case Reports, Clinical Trial Protocol | 68 |
| #11 | #6 NOT #5 | Books and Documents, Case Reports, Clinical Trial Protocol, Comment | 92 |
| #12 | #6 NOT #5 | Books and Documents, Case Reports, Clinical Trial Protocol, Comment, Editorial | 95 |
| #13 | #6 NOT #5 | Books and Documents, Case Reports, Clinical Trial Protocol, Comment, Editorial, Guideline | 101 |
| #14 | #6 NOT #5 | Books and Documents, Case Reports, Clinical Trial Protocol, Comment, Editorial, Guideline, Meta-Analysis | 165 |
| #15 | #6 NOT #5 | Books and Documents, Case Reports, Clinical Trial Protocol, Comment, Editorial, Guideline, Meta-Analysis, Review | 604 |
| #16 | #6 NOT #5 | Books and Documents, Case Reports, Clinical Trial Protocol, Comment, Editorial, Guideline, Meta-Analysis, Review, Systematic Review | 641 |
| #17 | #7 NOT #16 |  | 3,358 |
| #18 | #7 NOT #16 | Adult: 19+ years | 528 |
| #19 | #17 NOT #18 |  | 2,830 |
| #20 | #17 NOT #18 | English | 2,710 |
| #21 | #17 NOT #18 | English, MEDLIINE | 2,292 |
| Combined Syntax: | | | |
| ((((("attention deficit disorder with hyperactivity"[MeSH Major Topic] OR ("ADHD"[Title] OR "ADDH"[Title] OR "attention deficit"[Title] OR "inattenti*"[Title] OR "hyperactiv*"[Title] OR "hyper activ*"[Title] OR "hyperkine*"[Title])) AND ("recogni*"[Title/Abstract] OR "identifi*"[Title/Abstract] OR "detect*"[Title/Abstract] OR "diagnos*"[Title/Abstract] OR "refer*"[Title/Abstract] OR "help seek*"[Title/Abstract] OR "treatment seek*"[Title/Abstract] OR "misdiagnos*"[Title/Abstract] OR "underrecogni*"[Title/Abstract] OR "underdiagnos*"[Title/Abstract] OR "underdetect*"[Title/Abstract]) AND ("child*"[Title/Abstract] OR "adolescen*"[Title/Abstract] OR "youth*"[Title/Abstract] OR "teen*"[Title/Abstract] OR "young"[Title/Abstract] OR "student*"[Title/Abstract]) AND ("barrier*"[Title/Abstract] OR "hinder*"[Title/Abstract] OR "challeng*"[Title/Abstract] OR "promot*"[Title/Abstract] OR "facilitat*"[Title/Abstract] OR "encourag*"[Title/Abstract] OR "determina*"[Title/Abstract] OR "predict*"[Title/Abstract] OR "factor*"[Title/Abstract] OR "reason*"[Title/Abstract] OR "disparit*"[Title/Abstract] OR "issue*"[Title/Abstract])) NOT ("endocrin*"[Title/Abstract] OR "cardi*"[Title/Abstract] OR "heart"[Title/Abstract] OR "thyroid*"[Title/Abstract] OR "urin*"[Title/Abstract] OR ("College"[Title] OR "universit*"[Title] OR "undergraduate"[Title] OR "higher education"[Title] OR "postsecondary"[Title] OR "adult*"[Title]))) NOT (((("attention deficit disorder with hyperactivity"[MeSH Major Topic] OR ("ADHD"[Title] OR "ADDH"[Title] OR "attention deficit"[Title] OR "inattenti*"[Title] OR "hyperactiv*"[Title] OR "hyper activ*"[Title] OR "hyperkine*"[Title])) AND ("recogni*"[Title/Abstract] OR "identifi*"[Title/Abstract] OR "detect*"[Title/Abstract] OR "diagnos*"[Title/Abstract] OR "refer*"[Title/Abstract] OR "help seek*"[Title/Abstract] OR "treatment seek*"[Title/Abstract] OR "misdiagnos*"[Title/Abstract] OR "underrecogni*"[Title/Abstract] OR "underdiagnos*"[Title/Abstract] OR "underdetect*"[Title/Abstract]) AND ("child*"[Title/Abstract] OR "adolescen*"[Title/Abstract] OR "youth*"[Title/Abstract] OR "teen*"[Title/Abstract] OR "young"[Title/Abstract] OR "student*"[Title/Abstract]) AND ("barrier*"[Title/Abstract] OR "hinder*"[Title/Abstract] OR "challeng*"[Title/Abstract] OR "promot*"[Title/Abstract] OR "facilitat*"[Title/Abstract] OR "encourag*"[Title/Abstract] OR "determina*"[Title/Abstract] OR "predict*"[Title/Abstract] OR "factor*"[Title/Abstract] OR "reason*"[Title/Abstract] OR "disparit*"[Title/Abstract] OR "issue*"[Title/Abstract])) NOT ("endocrin*"[Title/Abstract] OR "cardi*"[Title/Abstract] OR "heart"[Title/Abstract] OR "thyroid*"[Title/Abstract] OR "urin*"[Title/Abstract] OR ("College"[Title] OR "universit*"[Title] OR "undergraduate"[Title] OR "higher education"[Title] OR "postsecondary"[Title] OR "adult*"[Title]))) AND ("pubmed books"[Filter] OR "case reports"[Publication Type] OR "clinical trial protocol"[Publication Type] OR "hascommenton"[All Fields] OR "editorial"[Publication Type] OR "guideline"[Publication Type] OR "letter"[Publication Type] OR "meta analysis"[Publication Type] OR "practice guideline"[Publication Type] OR "review"[Publication Type] OR "systematic review"[Filter]))) NOT ((((("attention deficit disorder with hyperactivity"[MeSH Major Topic] OR ("ADHD"[Title] OR "ADDH"[Title] OR "attention deficit"[Title] OR "inattenti*"[Title] OR "hyperactiv*"[Title] OR "hyper activ*"[Title] OR "hyperkine*"[Title])) AND ("recogni*"[Title/Abstract] OR "identifi*"[Title/Abstract] OR "detect*"[Title/Abstract] OR "diagnos*"[Title/Abstract] OR "refer*"[Title/Abstract] OR "help seek*"[Title/Abstract] OR "treatment seek*"[Title/Abstract] OR "misdiagnos*"[Title/Abstract] OR "underrecogni*"[Title/Abstract] OR "underdiagnos*"[Title/Abstract] OR "underdetect*"[Title/Abstract]) AND ("child*"[Title/Abstract] OR "adolescen*"[Title/Abstract] OR "youth*"[Title/Abstract] OR "teen*"[Title/Abstract] OR "young"[Title/Abstract] OR "student*"[Title/Abstract]) AND ("barrier*"[Title/Abstract] OR "hinder*"[Title/Abstract] OR "challeng*"[Title/Abstract] OR "promot*"[Title/Abstract] OR "facilitat*"[Title/Abstract] OR "encourag*"[Title/Abstract] OR "determina*"[Title/Abstract] OR "predict*"[Title/Abstract] OR "factor*"[Title/Abstract] OR "reason*"[Title/Abstract] OR "disparit*"[Title/Abstract] OR "issue*"[Title/Abstract])) NOT ("endocrin*"[Title/Abstract] OR "cardi*"[Title/Abstract] OR "heart"[Title/Abstract] OR "thyroid*"[Title/Abstract] OR "urin*"[Title/Abstract] OR ("College"[Title] OR "universit*"[Title] OR "undergraduate"[Title] OR "higher education"[Title] OR "postsecondary"[Title] OR "adult*"[Title]))) NOT (((("attention deficit disorder with hyperactivity"[MeSH Major Topic] OR ("ADHD"[Title] OR "ADDH"[Title] OR "attention deficit"[Title] OR "inattenti*"[Title] OR "hyperactiv*"[Title] OR "hyper activ*"[Title] OR "hyperkine*"[Title])) AND ("recogni*"[Title/Abstract] OR "identifi*"[Title/Abstract] OR "detect*"[Title/Abstract] OR "diagnos*"[Title/Abstract] OR "refer*"[Title/Abstract] OR "help seek*"[Title/Abstract] OR "treatment seek*"[Title/Abstract] OR "misdiagnos*"[Title/Abstract] OR "underrecogni*"[Title/Abstract] OR "underdiagnos*"[Title/Abstract] OR "underdetect*"[Title/Abstract]) AND ("child*"[Title/Abstract] OR "adolescen*"[Title/Abstract] OR "youth*"[Title/Abstract] OR "teen*"[Title/Abstract] OR "young"[Title/Abstract] OR "student*"[Title/Abstract]) AND ("barrier*"[Title/Abstract] OR "hinder*"[Title/Abstract] OR "challeng*"[Title/Abstract] OR "promot*"[Title/Abstract] OR "facilitat*"[Title/Abstract] OR "encourag*"[Title/Abstract] OR "determina*"[Title/Abstract] OR "predict*"[Title/Abstract] OR "factor*"[Title/Abstract] OR "reason*"[Title/Abstract] OR "disparit*"[Title/Abstract] OR "issue*"[Title/Abstract])) NOT ("endocrin*"[Title/Abstract] OR "cardi*"[Title/Abstract] OR "heart"[Title/Abstract] OR "thyroid*"[Title/Abstract] OR "urin*"[Title/Abstract] OR ("College"[Title] OR "universit*"[Title] OR "undergraduate"[Title] OR "higher education"[Title] OR "postsecondary"[Title] OR "adult*"[Title]))) AND ("pubmed books"[Filter] OR "case reports"[Publication Type] OR "clinical trial protocol"[Publication Type] OR "hascommenton"[All Fields] OR "editorial"[Publication Type] OR "guideline"[Publication Type] OR "letter"[Publication Type] OR "meta analysis"[Publication Type] OR "practice guideline"[Publication Type] OR "review"[Publication Type] OR "systematic review"[Filter]))) AND "adult"[MeSH Terms])) AND ((medline[Filter]) AND (english[Filter])) | | | |

**Table 4**

*A Summary of the Inclusion and Exclusion Criteria for the Systematic Review.*

|  | Inclusion criteria | Exclusion criteria |
| --- | --- | --- |
| Types of studies | - All original research articles using all types of methodologies and designs. - Grey literature, unpublished literature, pre-prints, dissertations, and theses. | - Papers that did not include any data, e.g., commentaries, opinions, editorials, book chapters, practice guidelines etc. - Review papers and case studies. - Articles with no full-text access. |
| Types of phenomena of interest | - Studies that explored child-level factors that influence the diagnosis of ADHD, where diagnosis is defined as any stage within the diagnostic process of ADHD, and child-level factors refer to any clinical characteristics and socio-demographic background of the children or adolescents with ADHD. - Studies that explored factors affecting ADHD care or service in general will be included if they indicated factors which are affecting identification and diagnosis of ADHD specifically. - Studies that explored factors on a higher level will be included if they are related to the characteristics, clinical factors or sociodemographic background of the children. | - Studies that only investigated barriers on a community, organisational, systemic and societal level. (Note. Country of residence will be considered as a societal-level factor) - Studies that focused on barriers to accessing care after ADHD diagnosis. - Studies that focused only on interventions to detect ADHD symptoms or behaviours without examining the barriers to ADHD diagnosis specifically. - Studies that only reported on differences in prevalence rates between different groups without addressing whether/ how these group characteristics relate to the detection and diagnosis of ADHD. - Studies that addressed the diagnosis of children with mental health difficulties broadly. - Studies that investigated the diagnostic criteria of ADHD rather than the diagnostic pathway of ADHD. - Studies that explored factors that are assumed to affect ADHD diagnosis, but did not make an explicit association between these factor(s) and the diagnosis of ADHD. |
| Population of interest | - Studies including individuals aged ≤18 years, with a clinical or research diagnosis of ADHD. - Studies with mixed-age populations will be included if it possible to extract data for individuals under 18 separately; if most participants were under 18; or if it is apparent that they were diagnosed before 18 years old. - Studies with a mixed-diagnosis sample will be included if the data for the ADHD group or ADHD-specific diagnostic subscales is reported separately. - Primary studies in all settings, with both clinical and/or community populations. - There will be no restrictions in the ADHD presentation, health status or co-morbidities of children with ADHD. | - Studies centred around adults aged ≥ 19 years or adult-onset ADHD, as previous research has suggested that the barriers to an ADHD diagnosis in adults differ substantially compared to children and adolescents (Ginsberg et al., 2014). - Studies that did not involve children and adolescents with a diagnosis of ADHD, or a diagnosable presentation of ADHD indicated with a validated ADHD-specific measure. - Studies that only focus on specific mental health condition other than ADHD, and/ or behaviour and/ or emotional problems more generally will not be included. |
| Other features of studies | - Studies reported in the English language. - No restrictions on country of study or sample sizes. - Studies with publication dates from 1968 to the date of the literature search. | - Studies not reported in the English language. - Studies published before 1968. |

*Data Extraction Proforma*

General information

- Title
- Authors
- Year of publication
- Country in which the study is conducted
- Study type
  - Non-randomised experimental study
  - Cohort study
  - Prospective longitudinal study
  - Case-control study (retrospective)
  - Cross-sectional analytical study
  - Analytical descriptive study e
  - Other
- Possible conflict(s) of interest for study authors

Aims and Hypotheses

- Aim(s)
- Hypothesis(es)

Methodology

*Recruitment Method*

- Sample source
  - Schools/ nurseries/ daycare
  - Outpatients/ clinics/ hospitals
  - Birth/ longitudinal cohorts
  - National databases/ registries
  - Other
- Sample source - context

*Participating Children Characteristics*

- Total number of child participants
- Age (e.g., mean, range, SD)
- Gender
- Race/Ethnicity
- Inclusion criteria
- Exclusion criteria
- Other relevant participants information/ characteristics

*Data Collection Method*

- Total data collection duration (start and end date)
- Data collection setting
  - Schools/ nurseries/ daycare
  - Outpatient/ clinics/ healthcare setting
  - Households
  - Virtual (phone, mail)
  - Unclear
  - Other

*Data collection procedures*

- Evidence of ADHD diagnosis/ ADHD diagnostic measure(s)
- Measure(s) of ADHD symptoms/ severity
- Measure(s) of other child clinical characteristics
- Socio-demographic measure(s)
- Other relevant data collected
- Other relevant information about data collected

*Data Analysis Method*

- Data analysis procedures
- Were other confounding variables controlled?
  - Yes
  - No
  - Unclear
- If yes, please provide details of what confounding variable(s) were controlled.

Results

- Child-related factor(s) in question
- Stage(s) within diagnostic process affected
- Direction of effect(s)
- Is/ are the effect(s) significant?
- Summary of relevant key findings
- Authors’ relevant conclusion/ discussion

Risk of bias information

- Strength(s)
- Limitation(s)

**Table 5**

*The Modified Version of the Risk of Bias Assessment tool for Non-randomised Studies (RoBANS; Kim et al., 2013).*

| 1. The selection of participants |
| --- |
| Selection biases caused by the inadequate selection of participants.  E.g., Are the comparison groups clearly defined, selected from the same population, or comparable? Are the measures that separate the groups reliable and valid? |
| 1. Confounding variables |
| Selection biases caused by the inadequate confirmation and consideration of confounding variables - the major confounding variables should be adequately confirmed AND considered/ adjusted for either during the design phase or analysis phase.  E.g., through participants matching or restrictions, stratification, or statistical adjustments etc. |
| 1. Measurement of child-level factors |
| Assessment or measurement biases caused by inadequate measurements of factors in question.  'Low risk' if data were obtained from trustworthy sources, reliable and valid instruments, or structured interviews; 'High risk' if data were obtained through non-trustworthy sources, like self-report methods, presence of inconsistencies in data collection methods, is a clear case of interviewer bias, or recall bias. |
| 1. Blinding of outcome measurements |
| Detection biases caused by the inadequate blinding of outcome assessments.  'Low risk' if the outcome assessment is blinded, or if blinding was not present, but its absence was judged to have no effect on the outcome measurements; 'High risk' if blinding was not performed or incomplete, and this lack of appropriate blinding appears likely to have affected the outcome measurements, e.g., diagnostic inaccuracies. |
| 1. Incomplete outcome data |
| Attrition or non-response biases caused by the inadequate handling of incomplete outcome data/ missing data. |
| 1. Selective outcome reporting |
| Reporting biases caused by the selective reporting of outcomes.  E.g., Are predefined or expected outcomes included in the study descriptions or experimental protocol? Are these expected outcomes fully reported? Are there incomplete reporting, or absence of reports on important outcomes that would be expected for studies in related fields? |

Supplemental File G

*The PRISMA 2020 Checklist (Page et al., 2021) for the Systematic Review.*

| **Section and Topic** | **Item #** | **Checklist item** | **Location where item is reported** |
| --- | --- | --- | --- |
| **TITLE** | | |  |
| Title | 1 | Identify the report as a systematic review. | p.1 |
| **ABSTRACT** | | |  |
| Abstract | 2 | See the PRISMA 2020 for Abstracts checklist. | N/A |
| **INTRODUCTION** | | |  |
| Rationale | 3 | Describe the rationale for the review in the context of existing knowledge. | p.7 |
| Objectives | 4 | Provide an explicit statement of the objective(s) or question(s) the review addresses. | p.8 |
| **METHODS** | | |  |
| Eligibility criteria | 5 | Specify the inclusion and exclusion criteria for the review and how studies were grouped for the syntheses. | p.9-10 + Supplementary Material 1 table 4 |
| Information sources | 6 | Specify all databases, registers, websites, organisations, reference lists and other sources searched or consulted to identify studies. Specify the date when each source was last searched or consulted. | p.9 + Supplementary material 1 table 2 |
| Search strategy | 7 | Present the full search strategies for all databases, registers and websites, including any filters and limits used. | Supplementary material 1 table 1, 2 and 3 |
| Selection process | 8 | Specify the methods used to decide whether a study met the inclusion criteria of the review, including how many reviewers screened each record and each report retrieved, whether they worked independently, and if applicable, details of automation tools used in the process. | p.10 |
| Data collection process | 9 | Specify the methods used to collect data from reports, including how many reviewers collected data from each report, whether they worked independently, any processes for obtaining or confirming data from study investigators, and if applicable, details of automation tools used in the process. | p.10 |
| Data items | 10a | List and define all outcomes for which data were sought. Specify whether all results that were compatible with each outcome domain in each study were sought (e.g. for all measures, time points, analyses), and if not, the methods used to decide which results to collect. | p.10 + Supplementary material 1 |
|  | 10b | List and define all other variables for which data were sought (e.g. participant and intervention characteristics, funding sources). Describe any assumptions made about any missing or unclear information. | p.10 + Supplementary material 1 |
| Study risk of bias assessment | 11 | Specify the methods used to assess risk of bias in the included studies, including details of the tool(s) used, how many reviewers assessed each study and whether they worked independently, and if applicable, details of automation tools used in the process. | p.10-11 + Supplementary material 1 table 5 |
| Effect measures | 12 | Specify for each outcome the effect measure(s) (e.g. risk ratio, mean difference) used in the synthesis or presentation of results. | N/A |
| Synthesis methods | 13a | Describe the processes used to decide which studies were eligible for each synthesis (e.g. tabulating the study intervention characteristics and comparing against the planned groups for each synthesis (item #5)). | N/A |
|  | 13b | Describe any methods required to prepare the data for presentation or synthesis, such as handling of missing summary statistics, or data conversions. | N/A |
|  | 13c | Describe any methods used to tabulate or visually display results of individual studies and syntheses. | N/A |
|  | 13d | Describe any methods used to synthesize results and provide a rationale for the choice(s). If meta-analysis was performed, describe the model(s), method(s) to identify the presence and extent of statistical heterogeneity, and software package(s) used. | N/A |
|  | 13e | Describe any methods used to explore possible causes of heterogeneity among study results (e.g. subgroup analysis, meta-regression). | N/A |
|  | 13f | Describe any sensitivity analyses conducted to assess robustness of the synthesized results. | N/A |
| Reporting bias assessment | 14 | Describe any methods used to assess risk of bias due to missing results in a synthesis (arising from reporting biases). | N/A |
| Certainty assessment | 15 | Describe any methods used to assess certainty (or confidence) in the body of evidence for an outcome. | N/A |
| **RESULTS** | | |  |
| Study selection | 16a | Describe the results of the search and selection process, from the number of records identified in the search to the number of studies included in the review, ideally using a flow diagram. | p.11 + Figure 1 |
|  | 16b | Cite studies that might appear to meet the inclusion criteria, but which were excluded, and explain why they were excluded. | p.12 |
| Study characteristics | 17 | Cite each included study and present its characteristics. | Table 1 + Supplementary material 2 |
| Risk of bias in studies | 18 | Present assessments of risk of bias for each included study. | Supplementary material 3 |
| Results of individual studies | 19 | For all outcomes, present, for each study: (a) summary statistics for each group (where appropriate) and (b) an effect estimate and its precision (e.g. confidence/credible interval), ideally using structured tables or plots. | N/A |
| Results of syntheses | 20a | For each synthesis, briefly summarise the characteristics and risk of bias among contributing studies. | N/A |
|  | 20b | Present results of all statistical syntheses conducted. If meta-analysis was done, present for each the summary estimate and its precision (e.g. confidence/credible interval) and measures of statistical heterogeneity. If comparing groups, describe the direction of the effect. | N/A |
|  | 20c | Present results of all investigations of possible causes of heterogeneity among study results. | N/A |
|  | 20d | Present results of all sensitivity analyses conducted to assess the robustness of the synthesized results. | N/A |
| Reporting biases | 21 | Present assessments of risk of bias due to missing results (arising from reporting biases) for each synthesis assessed. | N/A |
| Certainty of evidence | 22 | Present assessments of certainty (or confidence) in the body of evidence for each outcome assessed. | N/A |
| **DISCUSSION** | | |  |
| Discussion | 23a | Provide a general interpretation of the results in the context of other evidence. | p.27-33 |
|  | 23b | Discuss any limitations of the evidence included in the review. | Throughout review + p.34-35 |
|  | 23c | Discuss any limitations of the review processes used. | p.35 |
|  | 23d | Discuss implications of the results for practice, policy, and future research. | p.36-37 |
| **OTHER INFORMATION** | | |  |
| Registration and protocol | 24a | Provide registration information for the review, including register name and registration number, or state that the review was not registered. | p.9 |
|  | 24b | Indicate where the review protocol can be accessed, or state that a protocol was not prepared. | p.9 |
|  | 24c | Describe and explain any amendments to information provided at registration or in the protocol. | p.11 |
| Support | 25 | Describe sources of financial or non-financial support for the review, and the role of the funders or sponsors in the review. | p.2 |
| Competing interests | 26 | Declare any competing interests of review authors. | p.2 |
| Availability of data, code and other materials | 27 | Report which of the following are publicly available and where they can be found: template data collection forms; data extracted from included studies; data used for all analyses; analytic code; any other materials used in the review. | p.2 |

*From:*  Page MJ, McKenzie JE, Bossuyt PM, Boutron I, Hoffmann TC, Mulrow CD, et al. The PRISMA 2020 statement: an updated guideline for reporting systematic reviews. BMJ 2021;372:n71. doi: 10.1136/bmj.n71

For more information, visit: <http://www.prisma-statement.org/>
