# Supplementary material for: Individual child factors affecting the diagnosis of attention deficit hyperactivity disorder (ADHD) in children and adolescents: a systematic review
Source: Eur Child Adolesc Psychiatry. 2024 Oct 7;34(5):1469–96. doi: 10.1007/s00787-024-02590-9 (PMC12122567; doi:10.1007/s00787-024-02590-9)
Supplement: Supplementary file 2 — Supplementary file2 (DOCX 48 kb) [file 787_2024_2590_MOESM2_ESM.docx]

**Supplementary Material 2: Detailed Characteristics of the Studies Included in the Review.**

| Reference | Country | Recruitment Source | Study Design | Number of ADHD children/ sample size | Diagnostic criteria/ tool + informant | Characteristics of ADHD children (gender, age range, ethnicity) | Statistical approach | ADHD diagnostic process under investigation | Individual factors investigated | Relevant aim of study/ scope of research |
| --- | --- | --- | --- | --- | --- | --- | --- | --- | --- | --- |
| Arruda et al., 2022 | Brazil | Schools from 87 cities in 18 Brazilian states. | Cross-sectional analytical study; Parents and teachers were interviewed using standardized questions. | 7114 children; 505 ADHD-diagnosed children; 277 ADHD-probable children. | - Parent-reported clinical ADHD diagnosis by a doctor or a professional.  - ADHD-probable: determined by the parent- and teacher-reported Brazilian version of the Multimodality Treatment Study scale (MTA-SNAP-IV; Mattos et al., 2006), which assesses the presence of all 18 symptoms of ADHD according to DSM-V criteria (APA, 2013). | - 5-18 years  - 59.6% 5-9 years; 32.3% 10-13 years; 8.1% 14-18 years  - 49.9% male  - 64.8% White; 31.3% Non-white; 3.9% non-respondents | Logistic regressions (Likelihood ratio and Wald tests) | Likelihood of a clinical diagnosis of ADHD and probable-ADHD | Income class  School type  Gender  Ethnicity  Population density  Age  School performance | To estimate the prevalence of parent-reported ADHD diagnosis, the prevalence of children with probable ADHD according to DSM-5 criteria, and identified potential predictors of these outcomes, to better understand community prevalence i.e., children identified in a representative population sample) and administrative prevalence (i.e., people receiving a clinical diagnosis in the population). |
| Arya et al., 2015 | India | Newly registered patients attending the Child and Adolescent Mental Health Service (CAMHS) outpatient department in India. | Cross-sectional analytical study; semi-structured interviews with parents. | 57 children with ADHD. | Children who fulfilled the DSM-IV-TR (APA, 2000) criteria for ADHD determined through semi-structured interviews with parents, consensus diagnosis made with at least 2 authors. | - 6-16 years  - 85.9% 6-12 years; 14% 13-16 years 8  - 70.2% male | Comparison analyses | Delay in contacting specialist services | Gender  Age  Urbanicity  SES  Subtype  Comorbidities  Family type | To assess the pathway of care in ADHD among patients attending the child and adolescent mental health service (CAMHS) of a tertiary care centre in India. |
| Bannett et al., 2021 | US | Packard Children’s Health Alliance (PCHA) – a large community-based paediatric primary health care network in the San Francisco Bay Area, affiliated with Stanford Children’s Health and Lucile Packard Children’s Hospital. | Retrospective analytical study; structured electronic health records (EHR) by all PCHA PCPs from October 1, 2015 to March 30, 2019. | 195 children with ADHD; 29,213 non-ADHD children. | Children who had at least 1 visit with an ICD-10 (WHO, 2013) text descriptor of ADHD diagnosis. | - 2-5 years  - 72.8% male  - race: 36.4% white; 11.8% Asian; 4.6% Black; 19% Other; and 28.2% unknown  - ethnicity: 54.9% non-Hispanic; 14.9% Hispanic; 30.3 unknown | Multivariate logistic regression | The presence and timings of a clinical diagnosis of ADHD | Comorbidities  Gender  Insurance type | To assess prevalence of documented ADHD diagnoses (symptoms and disorder) among young children aged 2−5 years and to identify patient factors associated with ADHD diagnosis and adherence to guidelines. |
| Barry et al. 2016 | US | Children from 40 schools within 5 school districts located in the central and southern regions of the state of Mississippi. | Cross-sectional analytical study; teacher-reported questionnaires and semi-structured telephone interview with parents. | 1044 children with ADHD. | A research diagnosis of ADHD derived from Vanderbilt AD/HD Diagnostic Teacher Rating Scale (VADTRS; Wolraich et al., 2013), which include a comprehensive list of the 18 diagnostic symptoms of the disorder derived directly from the DSM (APA, 2013). | - mean age = 8.7 years (SD = 1.6)  - 66.4% male  - 64.1% White, 31.7% Black, 1.8% Hispanic/Latino, 0.9% Asian, 0.5%, Native American, 0.2% Native Hawaiian, 0.9% not reported | Correlations and multiple comparisons analyses | Referral and parental recognition of ADHD | Race  SES  Gender  Age  ADHD symptoms  ODD/CD symptoms  Anxiety/ depression symptoms  Teacher-rated classroom impairment | To improve early identification of children at risk for attention-deficit/ hyperactivity disorder (ADHD) and communicate these concerns to parents, recommending that they contact their child’s primary care provider (PCP). |
| Bax et al., 2019 | US | Multi-site elementary school-based sample; children from elementary schools from 5 different school districts from either Oklahoma or South Carolina. | Cross-sectional analytical study; secondary data from two separate but related studies: the Attention-Deficit Hyperactivity Disorder Prevalence Study, and the Tics/Attention-Deficit Hyperactivity Disorder Prevalence Study; both studies with structured interviews with parents and teachers. | 1068 children; 245 ADHD-diagnosed children; 259 ADHD-probable children. | - Parent-reported ADHD by a doctor or health professional.  - ADHD-probable: parent and teacher reports of ADHD symptom counts during interviews, with a minimum 6 DSM-IV-TR symptoms (APA, 2000) and a minimum 4–5 symptoms per VADTRS (Wolraich et al., 2013). | - 5-13 years  - 51.9% male  - 28.6% Black; 4.9% American Indian; 51.3% White; 10.4% Hispanic; 4.7% Other | Multivariate logistic regression | The likelihood of a clinical diagnosis of ADHD using the ADHD-probable group only | SES  Insurance  Race/ethnicity  Single parenthood  Gender  Urbanicity | To examine the relationships between demographic risk factors and ADHD diagnostic indicators, treatment, and access to care in a representative, community-based sample of elementary school-aged children. The study’s school-based samples allow for inclusion of children with ADHD who were not clinically diagnosed and those without overt signs of ADHD, which allows for a more accurate assessment of the relationship between sociodemographic factors (race/ethnicity, SES) and ADHD access-to-care rates. |
| Bonati et al., 2019 | Italy | Children requiring a visit to one of the Child and Adolescent Neuropsychiatric Services (CANPS) network of 18 regional specialized ADHD reference centres in the Lombardy region for a diagnostic evaluation. | Retrospective analytical study; medical records in stored ADHD registry database. | 2262 children; 1553 ADHD-diagnosed children. | Children diagnosed through a rigorous 7-step diagnostic evaluation; approved by all involved specialized clinicians to have met the DSM-IV-TR (APA, 2000) criteria for an ADHD diagnosis. | - 5-17 years (median age = 9)  - 85% males | Generalized linear model analyses | ADHD diagnostic evaluation pathway duration | Age  Gender  Psychiatric comorbidities  Chronic medical comorbidities  Clinician-rated ADHD severity | To examine waiting times for ADHD assessment in the 18 Regional ADHD centers, their variables, and inter-center differences for children and adolescents enrolled in the Registry between January 2013 and December 2017 for suspected ADHD. |
| Bonati et al., 2018 | Italy | Children requiring a visit to one of the CANPS network of 18 regional specialized ADHD reference centres in the Lombardy region for a diagnostic evaluation or treatment. | Retrospective longitudinal study; medical records in stored ADHD registry database. | 2856 ADHD-diagnosed children. | Children diagnosed through a rigorous 7-step diagnostic evaluation; approved by all involved specialized clinicians to have met the DSM-IV-TR (APA, 2000) criteria for an ADHD diagnosis. | - 6-17 years (mean age = 9.3 years; SD = 2.5)  - 86% males | Comparison analyses | The presence of a clinical diagnosis of ADHD | Relative age | To confirm whether relative age is associated with ADHD diagnosis, with or without comorbidities, and to investigate whether relative age is associated with ADHD type and severity, and if this age relationship is in common with other neurodevelopmental disorder. |
| Bussing et al., 2003 | US | Elementary school students in kindergarten through fifth grade during the 1998-99 academic year in a North Central Florida public school district. | Cross-sectional analytical study; parent and teacher questionnaires, and home interviews with parents. | 389 children with ADHD. | A research diagnosis for children who scored greater than 2 SDs above the norm on the parent and teacher SNAP-IV (Swanson, 1992) screening measure; or parent-reported clinical diagnosis of ADHD or were currently under treatment for ADHD. | - mean age = 7.8 years (SD = 1.8)  - 52% male  - 48% Caucasian; 52% African American | Comparisons and multivariate logistic regression | Parental problem recognition, professional evaluation, and clinical diagnosis of ADHD | Gender  Age  Race  SES  Insurance  Parent-rated severity | Using a district-wide sample of elementary school students at high risk for ADHD to examine whether help-seeking steps (i.e., recognising a child problem, seeking an evaluation, obtaining a professional diagnosis, and securing ADHD treatment) vary by child gender or ethnicity, after controlling for SES and enabling and need variables; and to seek to describe barriers to service use from the perspective of parents whose children meet DSM-IV criteria for ADHD and have not utilised any mental health services in the prior 12 months. |
| Chen et al., 2021 | Taiwan | All individuals (n = 9,548,393) born In Taiwan between September 1, 1984, and August 31, 2008 (aged 3-17 years between 2001 and 2011), extracted from the National Health Insurance Program (NHIP) in Taiwan, which has been implemented since 1995 to provide mandatory comprehensive medical care coverage to all civilian residents. | Retrospective analytical study; the National Health Insurance Research Database (NHIRD) between September 1, 2001 and August 31, 2011. | 149,384 children diagnosed with ADHD. | Children who were given an ICD-9-CM (WHO; 1992) code of ADHD (314.XX) at least twice by  board-certified psychiatrists in their medical records to ensure diagnostic validity. | - 3-17 years old  - 50.8% male | Poisson regression models with a robust error variance | The likelihood of a clinical diagnosis of ADHD | Urbanicity  Relative age | To clarify the impact of relative age in a school year with the diagnoses of ADHD, DD, anxiety disorder, and depressive disorder in Taiwan. |
| Chen et al., 2016 | Taiwan | The study cohort comprised of approximately 4.3% individuals aged 3-17 years in Taiwan between September 1, 1997 to August 31, 2011, randomly selected from the NHIP. | Retrospective analytical study; the 1997 – 2011 NHIRD. | ~8714 children diagnosed with ADHD. | Children who were given an ICD-9-CM (WHO, 1992) code of ADHD (314.XX) at least twice by  board-certified psychiatrists in their medical records. | 4-17 years old | Logistic regression | The likelihood of a clinical diagnosis of ADHD | Relative age | To explore the effect of relative age on the diagnosis and treatment of ADHD in a large population-based sample of subjects in Taiwan, to investigate whether the impact of relative age is exclusive to western countries, or is also present in Asian countries, such as Taiwan. |
| Coker et al., 2016 | US | All fifth-grade students in regular classrooms from 118 random samples of public schools from around Birmingham, Alabama; Los Angeles County, California; and Houston Texas. | Prospective longitudinal study; parent- and child- interviews at 3 waves (5^th^, 7^th^ and 10^th^ grade) at the home, a study center, or another preferred location. | 4297 children; 8% with clinical levels of ADHD symptoms and 8% diagnosed with ADHD. | Parent-reported diagnosis of ADHD by a physician or health professional in wave 1 and/or wave 3 (ADHD diagnosis was not asked in wave 2). | - Fifth grade at first data collection (median age = 11 years)  - 51.1% male  - 29.1% African-American; 44.4% Latino; 22.1% White | Comparisons and logistic regression (with both unadjusted and adjusted odds) | The likelihood of the presence of a clinical diagnosis of ADHD | Race/ ethnicity | To help address the question of whether the disparity in ADHD diagnosis and medication treatment is more likely due to an underdiagnosis or undertreatment of African-American and Latino children or an overdiagnosis or overtreatment of white children. |
| Dizdarevic et al. 2022 | Bosnia and Herzegovina | Two separate source of school-aged children from the five largest Cantons in Federation BIH (Sarajevo Canton, Tuzla Canton, Zenica-Doboj Canton, Hercegovina-Neretva Canton, and Bihac Canton) - students from the 100 selected schools, or registered patients from the 5 major hospitals in the 5 Cantons. | Cross-sectional analytical study; parent- questionnaires, distributed through the 100 schools; questionnaires sent and filled by the 5 major hospitals. | 1935 children with completed parent-questionnaires; 97 children with clinical levels of ADHD symptoms;  138 children clinically diagnosed with ADHD. | - A confirmed medical diagnosis of ADHD provided by questionnaires filled by hospital staff.  - ADHD-probable: examined with Attention Deficit/ Hyperactivity Disorder Test (ADHDT; Gilliam, 1995), which is based on the DSM-IV (APA, 2000) criteria, using parent-reported symptoms. | - 6-15 years (mean age = 10.2; SD = 2.1)  - children with completed parent-questionnaires: 45.2% male  - clinically-diagnosed ADHD children: 63.8% male | Descriptive and comparison analyses | The presence of a clinical diagnosis of ADHD and probable-ADHD | Gender | To examine the differences in the prevalence rate of clinically confirmed ADHD cases and parent-reported ADHD cases in a large sample of children in Bosnia and Herzegovina (BIH); and to examine whether there are differences in ADHD prevalence in relation to child’s gender. |
| Elder, 2010 | US | Children who are living in states with state-wide kindergarten eligibility cut-off dates, from the Early Childhood Longitudinal Study, Kindergarten Class of 1998–1999 (ECLS-K), a nationally representative cohort of US children who entered kindergarten in the fall of 1998. | Population-based cohort study; parent and teacher interviews at 6 time intervals from 1999 – 2007 (kindergarten to eighth grade). | 11,784 children; 6.4% diagnosed with ADHD. | Parent-reported formal diagnosis of ADHD in any waves as of Spring 2007, during interviews. | - mean primary school starting age = 5.4 years (SD = .4)  - 8.4% non-Hispanic White; 5.1% non-Hispanic Black; 4.1% Hispanic | Local linear regression models with discontinuity estimates | The likelihood of a diagnosis of ADHD | Relative age | To investigate the role that subjective comparisons across children play in ADHD diagnoses by assessing whether children who are young relative to their classmates in school are disproportionately diagnosed with and eventually treated for ADHD; and to analyse the relationship between a child’s age relative to his classmates and both teacher- and parent-reported assessments of ADHD symptoms. |
| Evans et al., 2010 | US | Children in states where there was a state-wide age at school start law in force, and who were born within 120 days of the school eligibility cutoff date in their state and year, drawn from the National Health Interview Survey (NHIS), an annual survey of roughly 60,000 households that collects data on the extent of illness, disease, and disability in the civilian, non-institutionalized population of the United States. | Cross-sectional analytical study; data from the 1997- 2006 NHIS database. | 35,343 children; 8.6% was diagnosed with ADD/ ADHD. | Parent-reported clinical diagnosis of ADHD by a doctor or professional (information on ADHD diagnosis has been included in the Sample Child Supplement within the NHIS since 1997). | - 7-17 years (mean age = 11.8)  - 50.7% male  - 64.2% White; 15.9% Black; 15.2% Hispanic; 4.7% other race/ ethnicity | Linear probability regression with discontinuity estimates | The likelihood of a clinical diagnosis of ADHD | Relative age | To examine whether relative age is a significant determinant of ADHD diagnosis and treatment. |
| Froehlich et al., 2007 | US | The National Health and Nutrition Examination Survey (NHANES), an annual multistage probability sample survey of the noninstitutionalized US population, including an oversample of minority populations. | Cross-sectional analytical study; 2001-2004 NHANES data, collected through telephone interviews with parents. | 3082 children; 222 ADHD-probable children. | - Parent-reported diagnosis of ADHD by a doctor or a health professional.  - ADHD-probable: the National Institute of Mental Health Diagnostic Interview Schedule for Children–IV (DISC-IV), a structured diagnostic interview instrument, was used to assess the presence of ADHD based on DSM-IV (APA, 2000) criteria. | - 8-15 years  - 51% male  - 14.7% African American; 12% Mexican American; 10.8% Other; 62.5% non-Hispanic White | Multivariable logistic regression | The likelihood of a clinical diagnosis of ADHD among ADHD-probable children | ADHD subtype  Age  Gender  Race/ ethnicity  Income poverty  Health insurance status | To investigate sociodemographic predictors of ADHD prevalence, recognition and treatment; and the likelihood of receiving a prior diagnosis and medication treatment among children who fulfil DSM-IV ADHD criteria. |
| Halldner et al., 2014 | Sweden | Individuals born 1940 or later, living and residing in Sweden during July 2005 – December 2009, were identified using Swedish Total Population, Migration and Cause of Death Register. To identify individuals with ADHD, the National Patient Register (NPR), which has almost full coverage of all psychiatric inpatient care in Swedan since 1973; and the Prescribed Drug Register (PDR), which contains individualized information on all prescribed and dispensed prescription drugs from all Swedish pharmacies since July 2005, were used. | Nested case-control study (10 controls for each ADHD case individuals); extracted ADHD caseness and parent-reported ADHD symptoms from NPR, PDR and the Child and Adolescent Twin Study in Sweden (CATSS). | 12,233 6-17 years old children born in November – February,  diagnosed with ADHD. | ADHD caseness was defined either as a diagnosis (ICD-10 hyperkinetic disorders: F90) in the NPR, which contains best-estimate diagnoses assigned by attending clinicians to patients assessed or treated at different levels of healthcare; or as one or more dispensed prescriptions of ADHD medications amphetamine, dexamphetamine, methylphenidate or atomoxetine in the PDR from July 2005 through December 2009. | 6-17 years | Logistic regression models | The likelihood of an ADHD diagnosis | Relative age | To investigate whether relative immaturity alter the rate of ADHD diagnoses and medication; if the effect of relative immaturity changes with age; and if relative immaturity alter parents’ perception of ADHD symptoms in their children. |
| Hlavaty, 2020 | US | Children who are referred for psychological evaluation at the ADHD Center for Evaluation and Treatment (ACET) at Cleveland Clinic Children’s Hospital. | Cross-sectional analytical study; semi-structured interviews with parents and children, direct child evaluation, parent- and teacher-reported questionnaires. | 1331 children diagnosed with ADHD. | An in-person diagnostic assessment was conducted at ACET, which involved children and their parents completing a semi-structured interview administered by a Ph.D. level clinician or Master’s level counsellor, social worker, or psychology trainee that were supervised by a PhD level clinician. A diagnosis of ADHD was granted based on DSM guidelines of symptom quantity and impairment | - 3-18 years (mean age = 9.1, SD = 3.6)  - 70.7% male  - Race: 75.8% Caucasian; 14.5% African American; 6% Biracial; 1.8% Asian; 0.1% Native American; 1.8% Other  - Ethnicity: 7.2% Hispanic; 92.8% non-Hispanic | Structural equation modelling | Age at ADHD diagnosis | Parent- and teacher-rated ADHD symptoms  Parent-rated impairment  Parent- and teacher-rated behavioural problems  IQ  Social functioning  Emotional intelligence | To describe and better understand patterns of impairment, risk, and potential protective or compensatory factors that may impact the timing of initial ADHD diagnosis in a large, clinic based sample of children seeking initial diagnosis of ADHD. |
| Hoang et al., 2019 | UK/ England | All children aged <19 years (353,774) registered within the 158 UK general practices who are members of the Royal College of General Practitioners (RCGP) Research and Surveillance Centre (RSC) network in 2016. | Cross-sectional analytical study; information from the RCGP RSC sentinel network database; parent and child details linked through household key. | 3470 children with ADHD. | Children coded with a diagnosis of ADHD in the RCGP RSC sentinel network database. | - <19 years old  - 81.3% female (95% CI = 80.0-82.6%)  - 9.1% non-White ethnicity (95% CI = 8.1- 10.1%) | Multivariate log-level linear regression | Age at ADHD diagnosis | Family size  Family structure  Urbanicity  SES | To describe the variations in the average age of ADHD diagnosis and prescribing of stimulant medications among general practices who are members of the RCGP and RSC network; and to identify child, parental, household and general practice factors that might account for these variations. |
| Huss et al., 2008 | Germany | 14,836 children and adolescents from the German health interview and examination survey for children and adolescents (KiGGS) who are aged between 3-17 years at data collection. | Cross-sectional analytical study; information from the KiGGS database, collected from May 2003 until May 2006 in 167 representatively-selected sample points all over Germany. | 667 ADHD-diagnosed children; 644 ADHD-probable children. | - Parent-reported clinical diagnosis of ADHD by a medical doctor or psychologist.  - Probable ADHD: children who reached a clinically significant score of >= 7 on the HI subscale of the SDQ and have not yet been given a diagnosis by a medical doctor or psychologist. | - 3-17 years  - ADHD-diagnosed children: 81.7% male  - ADHD-probable children: 63.0% male | Binary logistic regression | The likelihood of the presence of a clinical diagnosis of ADHD and probable-ADHD | Gender  SES | To present distributions and odds ratios for both the lifetime prevalence of parent-reported professional ADHD diagnosis, as well as potential ADHD cases (symptom-based) for German children and adolescents from 3-17 years in age according to gender, age, SES and history of migration. |
| Karlstad et al., 2017 | Norway | The study population included all children born in Norway during 1998–2006 registered in the National Registry as resident in Norway at age six. | Population-based cohort study; various information was extracted from the National Registry and by linking it with four national registers. Information on ADHD diagnoses was obtained from two separate sources: the Norwegian Patient Registry (NPR), which holds information from specialist healthcare services from 2008 onwards;  and the Norwegian Directorate of Health reimbursement database for GPs, which holds information on consultations in primary healthcare from 2006 onwards. | 509,827 children; 17,105 children diagnosed with ADHD by specialists; 16,284 children diagnosed with ADHD by GP. | - Specialist ADHD diagnosis: an ICD-10 code F90 ‘Hyperkinetic disorder’ in the NPR.  - GP ADHD diagnosis: the International Classification of Primary Care 2nd edition (ICPC-2) code P81 ‘Hyperkinetic disorder’ in the Norwegian Directorate of Health reimbursement database for GPs. | - 6-14 years  - whole sample population: 51.2% male  - children with specialist ADHD diagnosis: 72.8% male  - children with GP diagnosis of ADHD: 72.4% male | Cox proportional hazards regression analyses | The likelihood of a diagnosis of ADHD | Relative age | To investigate whether calendar month of birth is associated with the risk of receiving ADHD medication or an ADHD diagnosis in Norway using large, population-based sample. |
| Klefsjö et al., 2021 | Sweden | Children with ADHD from 6 Child and Adolescent Psychiatric outpatient care units (CAPs) in western Sweden (Västra Gotaland region), who during the year of 2015 at the age of 17 years, randomly identified by the health care computer system used for registration of all daily contacts. | Retrospective case-control study; medical records in the health care computer system, collected on 30^th^ June 2016. | 100 children diagnosed with ADHD. | An ADHD diagnosis in the medical records in at least one registered visit in any of the six included CAPS. | - 17 years  - 50% male | Comparison analyses | Referral (referral reasons and source) and diagnostic process (age at first visit, age at first diagnosis, number of visits before receiving diagnosis, time span between first visit and diagnosis) | Gender | To examine if there are any gender differences in the diagnostic procedure and in received treatments prior to and after the ADHD diagnosis, in Swedish outpatient Child and Adolescent Psychiatric Clinic. |
| Layton et al., 2018 | US | 407,846 children born from 2007 through 2009 in the 18 states that use the September 1 cut-off for school entry, from the Truven Health MarketScan Research Database, a large health insurance claims database that contains individual-level information on more than 80 million enrollees in all U.S. states from approximately 100 commercial payers and self-insured corporations, not including Medicaid claims. | Cross-sectional analytical study; deidentified data extracted from insurance claims in the Truven Health MarketScan Research Database. | 71,672 children in analytic sample; 534 children diagnosed with ADHD. | A diagnosis of ADHD was based on ICD-9 code 314.01 (attention deficit disorder with hyperactivity) or 314.00 (attention deficit disorder without hyperactivity), or on the basis of any prescription filled for a stimulant in any insurance claim for a health care encounter between the child’s date of birth and December 2015 (the end of study period). | 4-17 years | Multivariable linear regression | The presence of a diagnosis of ADHD | Relative age | To study whether the rates of ADHD diagnosis and treatment differ among otherwise similar children who have been arbitrarily assigned to different grade cohorts. |
| Madsen et al., 2018 | Denmark | 51,527 children from the Danish National Birth Cohort (DNBC), a nationwide cohort study. The recruitment of participants took place at the first antenatal visit to the general practitioners throughout 1996–2003, and follow-up questionnaires were completed in the child’s 7^th^ year. | Birth cohort study; the DNBC follow-up questionnaires, completed by primary caregivers, either through the internet or on paper; and linked data from Danish national registries, including: the Danish National Patient Register, the Danish Psychiatric Central Research Register and the Danish National Prescription Registry. | 1373 ADHD-diagnosed children; 653 ADHD-probable children with the absence of a registered ADHD diagnosis. | - Children who were registered with a diagnosis of ADHD or had redeemed ADHD medication identified from the 3 Danish national registries.  - ADHD-probable: determined by a predictive algorithm (Goodman et al., 2004) using parent-reported SDQ-DEN (Niclasen et al., 2012). | - mean age at the end of follow-up = 12.5 years (SD = 1.4)  - total sample: 51.2% male  - ADHD-diagnosed children: 79% male  - ADHD-probable children but not diagnosed: 67% male | Multiple logistic regression | Clinical diagnosis of ADHD vs. ADHD-probable children with an absent of ADHD diagnosis | Gender  SES  Family status | To estimate the number of children with positive parent-reported SDQ ADHD behaviour at age 7 and absence of recorded ADHD diagnosis up till adolescence; and to investigate whether socio-demographic and other SDQ characteristics of this group differed from the children diagnosed with ADHD during follow-up. |
| Morgan et al., 2014 | US | Children enrolled in the Early Childhood Longitudinal Study Birth Cohort (ECLS-B), a longitudinal cohort study representative of US children born in 2001, at the 60-month assessment. | Birth cohort study; birth records, parent interviews, and direct child assessments at 4 time intervals between 9 and 60 months. | 6550 children; ~150 children with ADHD. | Parent-reported formal diagnosis of ADHD by a doctor since the child turned 4 years old in an interview. | - Mean age at the 60-month assessment = 64.7 months (SD = 3.8)  - 61.2% male  - 53.7% White; 13.9% Black; 25.1% Hispanic; 7.2% other race/ethnicity | Multiple logistic regression models | The likelihood of an ADHD diagnosis, and being in the lowest 9.2% of ADHD-related behavioural functioning | Race/ethnicity  Primary language  Gender  SES  Family structure  Cognitive functioning  Health insurance coverage | To identify risk factors for ADHD diagnosis by age five; to estimate the magnitude of any observed disparities among otherwise similar children, by controlling for many additional background characteristics at 24 months; and to examine whether population subgroups at greater or lesser risk of receiving ADHD diagnosis were more or less likely to display ADHD behavioural risk indicators (e.g., inattention, impulsivity). |
| Morgan et al., 2013 | US | Kindergarten children from the ECLS-K, a nationally representative cohort of US children who entered kindergarten in the fall of 1998. | Population-based cohort study; parent interviews and parent- and teacher-reported questionnaires, and direct child assessments at 6 data collection time intervals from 1999 – 2007 (kindergarten to eighth grade). | 17,100 children; 6.8% children diagnosed with ADHD. | Parent-reported formal diagnosis of ADD, ADHD or hyperactivity by a professional, during interviews in any data collection waves. | - mean age at first data collection = 68.5 months (SD = 4.4)  - 51.2% male  - ∼19% Hispanic; ~16% non-Hispanic African American; ~57% non-Hispanic white; ~8% other races/ethnicities | Discrete-time logistic (hazard) regression models | The likelihood of an ADHD diagnosis | Gender  Race/ethnicity  Primary language  Health insurance  Externalising problem behaviour  Academic achievements  SES | To investigate the onset and over-time dynamics of race/ethnic disparities in ADHD diagnosis across early and middle childhood in the US by extensively adjusting for plausible confounding factors (e.g., lower SES and lower behavioural and academic functioning). |
| Morgan et al., 2022 | US | Elementary school children participating in the Early Childhood Longitudinal Study: Kindergarten Class of 2010-2011 (ECLS-K: 2011), a nationally representative cohort of US children who entered kindergarten in the fall of 2011. | Population-based cohort study; data were collected in the spring of each grade (first to fifth grade) using parent and teacher ratings of children’s behavioural functioning, as well as by independently assessed measures of children’s academic and executive functioning. | 1070 children diagnosed with ADHD. | Parent-reported formal diagnosis of ADHD or ADD by a health professional between first and fifth grade, during interviews any survey waves. | 61% White; 39% other races/ ethnicities | Comparisons and multivariate logistic regression | The risk of being above-average in various intelligence and behavioural variables prior to ADHD children’s initial ADHD diagnosis (as a proxy of overdiagnosis); and the likelihood of being diagnosed with ADHD among children displaying above-average intelligence and behavioural variables the year prior | Race/ethnicity  SES | Using a population-based cohort to estimate the likelihood of ADHD overdiagnosis and overtreatment, as well as the sociodemographic disparities among U.S. schoolchildren who appeared unlikely to meet criteria for ADHD diagnosis and treatment based on independently administered measures of behavioral, academic, or executive functioning. |
| Morrow et al., 2012 | Canada | Children between the ages of 6 and 12 years any time during the study period of Dec 1, 1997 to Nov 30, 2008, and who have been registered in the provincial health plan for at least one year before their entry into the cohort. Children entered the cohort when the latest of the following events occurred: their sixth birthday, the start of the study period or having been covered by the provincial health plan for one year. | Retrospective analytical study; linked national data from provincial administrative health databases for prescription drugs (PharmaNet), physician services (Medical Services Plan) and hospital admissions (Canadian Institute for Health Information Discharge Abstracts Database), from 1997 – 2008. | 937,943 children. | ADHD diagnosis recorded in the national health databases. | - 6-12 years (mean age = 7.8)  - 51.3% male | Comparison and Cochran–Armitage trend test | The presence of an ADHD diagnosis | Relative age | To explore the influence of relative age on the diagnosis and treatment of ADHD in a large sample of children with data from a nearly universal public health plan in British Columbia, Canada. |
| Mowlem et al., 2019 | Sweden | Children from The Child and Adolescent Twin Study in Sweden (CATSS), an ongoing prospective longitudinal cohort twin study that targets all twins in Sweden born since 1992, who were assessed at age 9 years as baseline. | Cross-sectional analytical study; data from the National Patient Register (NPR), and a telephone interview with parents, no more than 1 month before or after the children’s 9^th^ birthday. | 19,804 children; 650 children clinically-diagnosed with ADHD; 2556 ADHD-probable children. | - Children were identified as having a clinical diagnosis of ADHD from the NPR if they had at least one record of inpatient or outpatient care coded for ADHD from 2001 to 2013.  - ADHD-probable: Based on DSM-5 (APA, 2013) ADHD symptom criteria using the parent-reported A-TAC questionnaire. | - 9 years at first data collection  - 50.64% males  - ~2.5:1 male-to-female ratio in clinically-diagnosed children  - ~1.8:1 male-to-female ratio in ADHD-probable children | Descriptives and multiple logistic regression models | The predictive association between various clinical characteristics and a clinical ADHD diagnosis, according to gender | Gender | To investigate sex differences in ADHD using a population-based and clinical sample in Sweden, by describing the severity of ADHD symptoms, conduct, and learning problems in males and females with and without clinically diagnosed ADHD, followed by examination of the ADHD symptom presentation; and to investigate whether the predictive associations of inattention, hyperactivity/impulsivity, conduct problems, and learning problems on being diagnosed and treated for ADHD differed in males and females. |
| O’Connor & McNicholas, 2020 | Ireland | Children from the Growing Up in Ireland – the National Longitudinal Study of Children (GUI), an ongoing longitudinal study that tracks the physical, psychological and social development of a nationally representative sample of Irish children. | Population-based cohort study; data from two waves of GUI were used - when the children were aged 9 (2007–2008) and 13 (2011–2012); data collected using parent, and teacher questionnaire and interviews; and direct child assessments. | 71 ADHD-diagnosed children; 582 ADHD-probable but undiagnosed children; 7915 non-ADHD controls. | - Children whose parent reported they held a formal clinical diagnosis of ADHD at age 9 from the GUI data.  - ADHD-probable: children with SDQ-HI scores of ≥ 9 (a conservative criterion) but without a parent-reported formal clinical diagnosis of ADHD. | - 9 years at first data collection  - ADHD-diagnosed children: 74.8% male  - ADHD-probable children: 72.1% male  - non-ADHD control group: 49.4% male | Comparisons analyses | ADHD-diagnosed vs. ADHD-probable but undiagnosed group | Gender  Single-carer household  Social class  Parental education  Equivalised income  Parent- and teacher- rated ADHD symptoms  General health  Conduct problems  Emotional symptoms  Peer relationships | To establish the socio-demographic profiles of 9-year-olds with diagnosed and undiagnosed ADHD symptoms in an Irish community sample; and to extract relevant information about these two cohorts’ health, service use and cognitive ability. |
| Oxley, 2000 | US | Students from three rural school districts in northeast Georgia and ranged in age from 6 to 11. | Cross-sectional analytical study; data from school records, psychological reports, and a social- emotional- behavioural rating scale completed by the participants’ teacher. | 155 children diagnosed with ADHD. | Children were evaluated and diagnosed by a certified school or licensed psychologist or psychiatrist according to the DSM-IV (APA, 2000) ADHD criteria, using a multi­modal, multi-informant diagnostic process that resulted in reliable clinical diagnoses. | - 6-11 years (mean age = 9.5)  - 81.9% male  - 87.7% Caucasians; 12.3% African Americans; no other minority groups were represented | Comparison analyses – MANOVA | Age at ADHD diagnosis | Subtype | To investigate the correlates associated with each of the two major ADHD subtypes, ADHD-CT and ADHD-PI, including age at diagnosis, and to determine whether significant differences exist. |
| Purper-Ouakil et al., 2007 | France | Children from the outpatient clinic of the Department of Child and Adolescent Psychopathology, a service with an evaluation unit for children with ADHD symptoms, of the University Hospital Robert Debre´ in Paris. | Retrospective analytical study; medical records and semi-structured interviews with parents. | 129 children with ADHD. | Children were confirmed to be diagnosed with ADHD using the French Kiddie-SADS lifetime version (Kiddie-SADS-PL; Mouren-Sime´on et al., 2002), a semi-structured diagnostic interview designed to assess current and past episodes of psychopathology in children and adolescents according to DSM-IV criteria (APA, 2000). | - 6-16 years (mean age = 125.5 months; SD = 34.1)  - 85.3% male | Comparisons and multivariate linear regression | Diagnostic delay (time between first consultation and definite diagnosis) | Co-morbid internalising and externalising disorders  Subtype  Gender  ADHD severity | To determine the mean diagnostic delay (i.e. the period between the first consultation with a health professional for impairing symptoms and the moment the parents are informed about the diagnosis) in a referred sample and to assess clinical variables potentially associated with the diagnostic process. |
| Root et al., 2019 | UK | Children in the UK Clinical Practice Research Datalink, which is a population-based large UK electronic primary care records database, with more than 700 general practices contributing to the dataset. | Population-based cohort study; electronic record data collected before January 3, 2017 in the UK Clinical Practice Research Datalink. | 1,039,430 children. | An ADHD diagnosis was primarily defined by first recorded relevant Read codes (as displayed in study’s eAppendix) in the electronic primary care records. | - 4-15 years (median age at study entry was 4.0 years; IQR = 4.0-5.0)  - Median age at ADHD diagnosis = 8.0 years (IQR = 6.7-9.7)  - 51.1% male | Multivariable Cox  proportional hazards regression models | The likelihood of an ADHD diagnosis | Relative age | To investigate the association between relative age in the school year and incidence of intellectual disability, attention-deficit/hyperactivity disorder, and depression. |
| Sayal et al., 2017 | Finland | All singleton live births in Finland between 1st January 1991 and 31st December 2004, recorded in Finnish National registries. | Birth cohort study; using records from two Finnish Nationwide registers, the Finnish Hospital Discharge Register (FHDR) and the Population Register Centre (PRC). | 6136 children with ADHD. | Children who were registered in the FHDR with a diagnosis of ADHD (International Classification of Diseases (ICD-10; WHO, 2013) codes: F90.0, F90.1, F90.8 and F90.9) by 31st December, 2011. | - 7-19 years (mean age = 9.4; SD = 2.4)  - 84.8% male | Poisson-regression model | The likelihood of an ADHD diagnosis | Relative age | To examine whether a relative age effect exists within a national context traditionally involving low rates of ADHD diagnosis and treatment, and if so, whether it operates more strongly at the younger end of the school age-range, if there is a temporal effect and if comorbid disorders play a role. |
| Sayal et al., 2010 | Great Britain | Children participated in the 1999 and 2004 British Child and Adolescent Mental Health Surveys (B-CAMHS), recruited through child benefit records. | Repeated cross-sectional analytical study; data collected from parental questionnaires and semi-structured interviews, teacher questionnaires, as well as questionnaires and semi-structured interviews with children aged 11 or over. | 176 children with ADHD in the 2004 survey; 238 in the 1999 survey. | ADHD diagnostic information in both surveys was based on the Development and Well-Being Assessment (DAWBA; Goodman et al., 2000) package that included a teacher questionnaire and a structured interview with the parent and child (if age 11 or older) that was administered by trained lay interviewers. Then, experienced clinicians reviewed all available information and assigned diagnoses of ADHD using DSM-IV criteria (APA, 2000) and hyperkinetic disorder using ICD-10 criteria (WHO, 2013). | - 2004 survey: 5-16 years (mean age = 10.3; SD = 3.2); 84% male  - 1999 survey: 5-15 years (mean age = 10.0; SD = 2.9); 81% male | Comparisons and multivariate logistic regression | Parental recognition and contact with professionals | Comorbid emotional or behavioural conditions  Parent- and teacher-rated impairment  Gender  Age  Single parenthood  House ownership  Symptom severity  General health | To examine predictors of recognition and service user for ADHD using cross-sectional national British data collected in 2004. |
| Sayal et al., 2006 | Great Britain | Children who participated in the 1999 B-CAMHS, recruited through child benefit records. | Cross-sectional analytical study; data collected from parental questionnaires and semi-structured interviews, teacher questionnaires, as well as questionnaires and semi-structured interviews with children aged 11 or over. | 232 children with ADHD. | Experienced clinicians reviewed all available diagnostic information based on the DAWBA package (Goodman et al., 2000) and assigned diagnoses of ADHD using DSM-IV criteria (APA, 2000) and hyperkinetic disorder using ICD-10 criteria (WHO, 2013). | - 5-15 years (mean age = 10.0; SD = 2.9)  - 81% male | Comparisons and multivariate logistic regression | Parental recognition and contact with professionals | Comorbid emotional or behavioural conditions  Parent- and teacher-rated impairment  Gender  Age  Single parenthood  House ownership  Symptom severity  General health | To investigate the roles of parent, teacher, child, and socio-demographic factors in determining parental recognition of problems and service use patterns in a large, nationally representative, epidemiological sample of children with ADHD. |
| Sayal et al., 2002 | England | - Referred clinical sample: 7-12-year-old children with a clinical diagnosis of ADHD, who had been referred by any Croydon GP to CAMHS in the previous year to study.  - Non-referred community sample: 5-11-year-old children registered with a random sample of 12 GPs in the outer London borough of Croydon, who were determined to have ADHD during screening. | Retrospective cross-sectional analytical study; medical records, parent and teacher questionnaires, and parental semi-structured interviews at home. | 127 children with ADHD; 58 ADHD-probable children; 40 children recognised with ADHD by GP. | - Children diagnosed by professionals in CAMHS after evaluation (taken from medical records).  - ADHD-probable: a score of 6 or above on the five ADHD items of both the parental and teacher-completed SDQ (Goodman, 1997), but not recognised with ADHD by GP. | - ADHD-probable children: mean age = 8.3 years (SD = 1.7)  - GP-recognised children: mean = 7.7 years (SD = 1.7) | Multivariate logistic regression | The likelihood of GP recognition and referral of ADHD in children | Parent-rated hyperactivity symptoms  Conduct problems  Emotional problems | To provide a quantitative description of the filters in the help-seeking pathway of children with pervasive hyperactivity through primary care and to investigate the relative contributions of child, parent and GP factors in determining service use. |
| Schwandt & Wuppermann, 2016 | Germany | Children aged 4 to 14 for the years 2008 through 2011 in the administrative medical claims records from all physicians registered with the German statutory health insurance (SHI), covering the universe of outpatient visits reimbursed by the SHI of all individuals. | Retrospective analytical study; data from the SHI administrative medical claims records, which contains information of all the children’s outpatient visits. | Roughly 7.2 million children; ~ 3.8% diagnosed with ADHD. | An ADHD diagnosis was defined as at least one recorded ADHD ICD-10 code during an outpatient visit, in two different quarters during a data year. | 4-14 years | Logistic regression with Ordinary Least Squares estimates | The likelihood of an ADHD diagnosis | Relative age | To show the existence and relevance of ADHD misdiagnoses around age cutoffs in one of the world's largest developed countries, Germany; and quantitatively explore potential mechanisms, relating average cutoff jumps to district-level characteristics. |
| Sikov et al., 2022 | US | Children aged 6-11 years who visited a large primary care paediatric clinic based in an urban safety-net teaching hospital for a routine health check between September 1, 2016 and August 31, 2017. | Cross-sectional analytical study; retrospective electronic medical records and parent-reported questionnaires. | 2,212 children; 264 children diagnosed with ADHD. | A patient was categorized as diagnosed with ADHD if they had any of the following ICD-10 codes on their active problem list: F90.0, F90.1, F90.2, F90.8, F90.9, F98.8, R41.840, and R46.3. | - 6-11 years (mean age = 8.6; SD = 1.7)  - 49.5% male  - 15.9% Hispanic/ Latino/ Spanish; 51.6% Non-Hispanic Black; 5.0% Non-Hispanic White; 23.7% Declined; 3.7% Other race/ ethnicity  - children diagnosed with ADHD: mean age = 9.1; SD = 1.6; 69.7% male; 17.8% Hispanic/ Latino/ Spanish; 58.7% Non-Hispanic Black; 7.6% Non-Hispanic White; 13.6% Declined; 2.3% Other race/ ethnicity | Multivariable logistic regression models | The likelihood of a clinical ADHD diagnosis | Primary language  Psychiatric comorbidities  Medical comorbidities  Gender  Insurance type  Age  Race/ ethnicity | To examine sociodemographic differences in ADHD diagnosis among school-age children; to understand whether there were sociodemographic differences in parent reporting of attention problems on universal screening, and whether variation in parent symptom reporting vs. other factors explained the sociodemographic differences found in ADHD diagnosis; to examine clinical factors and care processes associated with diagnostic disparities, including other diagnoses and documentation of diagnostic questionnaire results. |
| Staniszewski, 1999 | US | Students in kindergarten through third grades attending Whit Davis Elementary school, in Clarke County School District, during the 1997-1998 school year. | Cross-sectional analytical study; school records and teacher questionnaires. | 115 children with ADHD; 21 children referred to specialist; 94 non-referred ADHD children. | - School records indicated that a physician, a psychiatrist, a licensed psychologist, or a school psychologist has determined that the children have met the criteria for diagnosis and/ or treatment of ADHD.  - non-referred ADHD-probable students: students who demonstrates ADHD-like behaviours according to the teacher-reported BASC-TRS, based on scores > 49 on either or both inattentive or hyperactivity scales, but who were not formally diagnosed or selected for referral. | - kindergarten to third grade  - 71.3% male | Comparison analyses | ADHD students referred to specialist services vs. non-referred ADHD-probable students | Gender  Relative age  Intelligence  Academic  Competence  Learning problems  Adaptability  Aggression  Conduct problems | To compare referred ADHD-like students to non-referred ADHD-like students in grades K-3 on gender, birthdate, above average intelligence (as perceived by teacher), academic competence in math, academic competence in reading, BASC-TRS T-scores in the areas of learning problems, adaptability, aggression and conduct problems, as well as risk factor score (0-8); and to examine the differences in those referred in K versus referred in 1st, 2nd, or 3rd grades. |
| Stevens et al., 2004 | US | Children 3 to 18 years of age who visited primary care providers in office-based settings or hospital outpatient clinics, documented in the 1995-2000 National Ambulatory Medical Care Survey (NAMCS) and National Hospital Ambulatory Medical Care Survey (NHAMCS). | Cross-sectional analytical study; medical records (NAMCS and NHAMCS). | ~26450 children; 902 children diagnosed with ADHD. | ADHD diagnosis of children was based on codes on medical records assigned by providers during visits,  with ICD-9 codes of 314.00 (attention deficit disorder without mention of hyperactivity) and 314.01 (attention deficit disorder with hyperactivity). | 3-18 years | Comparisons and logistic regressions | The likelihood of an ADHD diagnosis | Race/ ethnicity  Age  Insurance type | To investigate ethnic and regional differences in childhood mental health care, focusing in particular on ADHD, using two large, nationally representative data sets containing information on office-based and hospital-base primary care visit for children 3 to 18 years of age. |
| Yamauchi et al., 2015 | Japan | Children with mental problems who visited one of the 16 leading hospitals in Japan that specialize in child psychiatric services for the first time between September 2008 and March 2009. | Cross-sectional analytical study; medical records and parent-reported questionnaire. | 387 children with ADHD. | Unclear | - mean age = 11.2 years (SD = 3.0)  - 88.3% male | Multivariate ordered logistic regression models | Time between initial parental concern and first visit to child psychiatric services | Age  Household income  Behavioural problems  Commute time  Parental education  Gender  Parent-rated impairment  Developmental delay | To examine how demographics, symptoms, and healthcare system factors affect the time between initial parental concerns about their child and the first visit to a hospital in Japan that offers child psychiatric services. |
