# Supplementary material for: Individual child factors affecting the diagnosis of attention deficit hyperactivity disorder (ADHD) in children and adolescents: a systematic review
Source: Eur Child Adolesc Psychiatry. 2024 Oct 7;34(5):1469–96. doi: 10.1007/s00787-024-02590-9 (PMC12122567; doi:10.1007/s00787-024-02590-9)
Supplement: Supplementary file 3 — Supplementary file3 (DOCX 35 kb) [file 787_2024_2590_MOESM3_ESM.docx]

**Supplementary Material 3: Risk of Bias Assessment Results.**

**Table 1**

*A Summary of the Results of the Methodological Quality of All Included Studies Using a Modified version of the Risk of Bias in Non-randomized Studies (RoBANS) Assessment.*

| Papers | Risk of Bias Domains | | | | | |
| --- | --- | --- | --- | --- | --- | --- |
|  | D1 | D2 | D3 | D4 | D5 | D6 |
| Arruda et al., 2022 | + | + | + | - | + | + |
| Arya et al., 2015 | + | - | + | - | + | + |
| Bannett et al., 2021 | + | ? | + | + | + | - |
| Barry et al. 2016 | + | - | - | - | + | + |
| Bax et al., 2019 | + | + | + | + | + | + |
| Bonati et al., 2019 | + | ? | + | + | ? | + |
| Bonati et al., 2018 | + | - | + | + | ? | + |
| Bussing et al., 2003 | + | + | + | + | + | - |
| Chen et al., 2021 | + | + | + | + | ? | + |
| Chen et al., 2016 | ? | + | + | + | ? | + |
| Coker et al., 2016 | + | + | + | - | + | + |
| Dizdarevic et al. 2022 | - | - | + | - | ? | ? |
| Elder, 2010 | + | + | + | - | + | + |
| Evans et al., 2010 | + | + | + | + | + | + |
| Froehlich et al., 2007 | + | + | + | + | + | + |
| Halldner et al., 2014 | + | ? | + | + | ? | + |
| Hlavaty, 2020 | + | + | ? | + | + | + |
| Hoang et al., 2019 | + | - | + | + | ? | + |
| Huss et al., 2008 | ? | - | + | - | ? | + |
| Karlstad et al., 2017 | + | + | + | + | ? | + |
| Klefsjö et al., 2021 | + | - | + | + | + | + |
| Layton et al., 2018 | + | + | + | - | + | + |
| Madsen et al., 2018 | + | + | + | - | ? | + |
| Morgan et al., 2014 | + | + | + | - | ? | - |
| Morgan et al., 2013 | + | + | - | + | ? | + |
| Morgan et al., 2022 | + | + | + | - | + | + |
| Morrow et al., 2012 | + | - | + | + | + | + |
| Mowlem et al., 2019 | + | - | + | - | - | + |
| O’Connor & McNicholas, 2020 | + | + | ? | - | + | + |
| Oxley, 2000 | - | ? | + | - | + | + |
| Purper-Ouakil et al., 2007 | - | - | + | + | + | - |
| Root et al., 2019 | + | + | ? | + | ? | + |
| Sayal et al., 2017 | + | - | + | + | ? | + |
| Sayal et al., 2010 | + | + | + | + | ? | - |
| Sayal et al., 2006 | + | + | + | + | ? | - |
| Sayal et al., 2002 | - | + | + | + | ? | + |
| Schwandt & Wuppermann, 2016 | + | + | + | + | ? | + |
| Sikov et al., 2022 | + | + | + | + | + | + |
| Staniszewski, 1999 | + | - | - | - | ? | + |
| Stevens et al., 2004 | + | ? | + | + | ? | + |
| Yamauchi et al., 2015 | ? | + | - | - | - | - |

*Note.* Judgements: + = Low risk of bias; - = High risk of bias; ? = Unclear risk of bias. Risk of bias domains: D1 = The selection of participants; D2 = Confounding variables; D3 = Measurement of child-level factors; D4 = Blinding of outcome measurements; D5 = Incomplete outcome data; D6 = Selective outcome reporting.

**Fig. 2**

Risk of Bias Graph: Review Authors’ Judgements about each Risk of Bias Items Presented in Percentages Across All Included Studies.
